# Thymoquinone, piperine, and sorafenib combinations attenuate liver and breast cancers progression: epigenetic and molecular docking approaches

**DOI:** 10.1186/s12906-023-03872-6

**Published:** 2023-03-04

**Authors:** Ashraf A. El-Shehawy, Alaa Elmetwalli, Ali H. El-Far, Sahar Abd El-Razik Mosallam, Afrah Fatthi Salama, Ahmad O. Babalghith, Mohammad A. Mahmoud, Hany Mohany, Mohamed Gaber, Tarek El-Sewedy

**Affiliations:** 1 Department of Chemistry, Faculty of Science, Kafrelsheikh University, Kafrelsheikh, 33516 Egypt; 2 Department of Clinical Trial Research Unit and Drug Discovery, Egyptian Liver Research Institute and Hospital (ELRIAH), Mansoura, Egypt; 3 Department of Biochemistry, Faculty of Veterinary Medicine, Damanhour University, Damanhour, 22511 Egypt; 4 Zoology Department, Women’s College for Arts, Science and Education, Ain Shams University, Cairo, Egypt; 5 Biochemistry Section, Chemistry Department, Faculty of Science, Tanta University, Tanta, 31527 Egypt; 6 Medical Genetics Department, College of Medicine, Umm Al-Qura University, Makkah, Saudi Arabia; 7 Chemistry Department, Faculty of Science, Tanta University, Tanta, 31527 Egypt; 8 Department of Applied Medical Chemistry, Medical Research Institute, Alexandria University, Alexandria, Egypt

**Keywords:** Thymoquinone, Piperine, Sorafenib, Epigenetic, Molecular docking

## Abstract

**Background:**

Traditional herbal medicine has been used for centuries to cure many pathological disorders, including cancer. Thymoquinone (TQ) and piperine (PIP) are major bioactive constituents of the black seed (*Nigella sativa*) and black pepper (*Piper nigrum)*, respectively. The current study aimed to explore the potential chemo-modulatory effects, mechanisms of action, molecular targets, and binding interactions after TQ and PIP treatments and their combination with sorafenib (SOR) against human triple-negative breast cancer (MDA-MB-231) and liver cancer (HepG2) cells.

**Methods:**

We determined drug cytotoxicity by MTT assay, cell cycle, and death mechanism by flow cytometry. Besides, the potential effect of TQ, PIP, and SOR treatment on genome methylation and acetylation by determination of DNA methyltransferase (*DNMT3B*), histone deacetylase (*HDAC3*) and miRNA-29c expression levels. Finally, a molecular docking study was performed to propose potential mechanisms of action and binding affinity of TQ, PIP, and SOR with DNMT3B and HDAC3.

**Results:**

Collectively, our data show that combinations of TQ and/or PIP with SOR have significantly enhanced the SOR anti-proliferative and cytotoxic effects depending on the dose and cell line by enhancing G2/M phase arrest, inducing apoptosis, downregulation of *DNMT3B* and HDAC3 expression and upregulation of the tumor suppressor, miRNA-29c. Finally, the molecular docking study has identified strong interactions between SOR, PIP, and TQ with DNMT3B and HDAC3, inhibiting their normal oncogenic activities and leading to growth arrest and cell death.

**Conclusion:**

This study reported TQ and PIP as enhancers of the antiproliferative and cytotoxic effects of SOR and addressed the mechanisms, and identified molecular targets involved in their action.

**Supplementary Information:**

The online version contains supplementary material available at 10.1186/s12906-023-03872-6.

## Background

Cancer is a global burden and is ranked as one of the major causes of death especially in low-income nations [1]. Globally, liver cancer is the 6^th^ most common and the third leading cause of cancer mortality (8.3%) after lung (18.0%) and colorectal (9.4%) cancer, with the highest incidence rates found in Asia and Africa [2]. On the other hand, worldwide, breast cancer has now exceeded lung cancer in incidence, with approximately 2.3 million new cases in 2020 and 685,000 deaths [3].

Chemotherapy is one of the major treatment patterns for cancer, used alone or in combination with other treatment modalities. Chemo-drugs kill cancer cells by interfering with the cell cycle regulating genes, therefore, inhibiting cell growth and proliferation, leading to cell death mainly by apoptosis [4–6]. Cancer patients receiving chemotherapy suffer from mild side effects, such as alopecia, constipation, and fatigue, to serious ones, such as sterility, cardiotoxicity, and pulmonary fibrosis. Therefore, reducing these side effects would contribute to a better lifestyle for these patients [7].

Sorafenib (SOR), doxorubicin, and cisplatin are the most used chemotherapeutic agents suitable for patients with liver and breast cancer. Sorafenib is a protein kinase inhibitor that is active against various protein kinases. In 2008, the US FDA approved SOR (NEXAVAR®) for treating patients with liver and advanced kidney cancers [8]. SOR has well-known anticancer potential (Supplementary File 1), as stated in the CTD database (http://ctdbase.org/). Also, it is used as a therapy for about 890 cancer clinical trials (Supplementary File 2), as recognized in the clinical trials database (https://clinicaltrials.gov/).

Natural products derived from plants have always been used to treat various diseases and most of the present anticancer drugs use active ingredients extracted from plant sources [9–13]. These compounds usually exert their effect through several mechanisms such as activation of apoptotic cell death, cell cycle arrest, and inhibition of angiogenesis [14].

Thymoquinone (TQ) is a major bioactive constituent of the black seed (*Nigella sativa*) [15], it has shown different anticancer activities through cell proliferation inhibition, and apoptosis induction in cancer cells, the possible mechanisms of TQ anticancer activity against various proliferative cancer cells were summarized in the review article by El-Far [16]. Interestingly, TQ was reported to have a significant selectivity against various malignant cells [17–21]. The anticancer potential and clinical trials of TQ were stated in Supplementary File 3 and Supplementary File 4, respectively. The taxonomic hierarchy of *Nigella sativa* L. is shown in Table 1 [22].

**Table 1:** Taxonomic hierarchy of *Nigella sativa*, *Piper nigrum*, and *Piper longum*

	***Nigella sativa* L**	***Piper nigrum* L**	***Piper longum* L**
Kingdom	*Plantae* – Plants	*Plantae*—Plants	*Plantae*—Plants
Subkingdom	*Tracheobionta*—Vascular plants	*Tracheobionta*—Vascular plants	*Tracheobionta*—Vascular plants
Superdivision	*Spermatophyta*—Seed plants	*Spermatophyta*—Seed plants	*Spermatophyta*—Seed plants
Division	*Magnoliophyta*—Flowering plants	*Magnoliophyta*—Flowering plants	*Magnoliophyta*—Flowering plants
Class	*Magnoliopsida* – Dicotyledons	*Magnoliopsida*—Dicotyledons	*Magnoliopsida*—Dicotyledons
Subclass	*Magnoliidae*	*Magnoliidae*	*Magnoliidae*
Order	*Ranunculales*	*Piperales*	*Piperales*
Family	*Ranunculaceae*—Buttercup family	*Piperaceae*—Pepper family	*Piperaceae*—Pepper family
Genus	*Nigella* L. – nigella	*Piper* L.—pepper	*Piper* L.—pepper
Species	*Nigella sativa* L. – black cumin	*Piper nigrum* L.—black pepper	*Piper longum* L.—Indian long pepper

Piperine (PIP) is the major bioactive alkaloid found in black (*Piper nigrum*), white and long pepper (*Piper longum*), it has several actions, including anti-inflammatory and anticancer properties [23]. The anticancer potential and clinical trials of TQ were stated in Supplementary File 5 and Supplementary File 6, respectively. Also, the taxonomic hierarchy of *Piper nigrum* [24] and *Piper longum* [25] is shown in Table 1.

This study examined the potential inhibitory effect of TQ, PIP, and SOR against human triple-negative breast cancer and hepatocellular carcinoma cells. Moreover, molecular targets and mechanisms involved in such activities were also investigated.

### Materials and methodsChemicals and reagents

TQ and PIP were obtained from Sigma-Aldrich Chemical Co. (St. Louis, Missouri, USA), and SOR was purchased from Cipla Ltd, India. All cell culture materials were obtained from Gibco (New York, New York, USA).

### Cell lines

Human hepatocellular carcinoma HepG2 and breast cancer MDA-MB-231 cells were supplied from American Type Culture Collection (ATCC). Cells were cultured in a complete DMEM medium and incubated at 37 °C in an atmosphere containing 5% CO_2_.

### Cytotoxicity assay

HepG-2 and MDA-MB-321 Cells were cultured at 15 × 10^3^ per well in a 96-well plate with 100 µl of complete fresh medium for 24 h before treatment with different concentrations of PIP (12.5–200 µM), TQ (25–400 µM) and SOR (6.25–100 µM) for 48 hs. Cell viability was measured by MTT as previously described [26], and the IC_50_ was calculated by nonlinear regression analysis of the dose–response curve in each cell line.

For the determination of IC_50_ values in the combination treatments of TQ and/or PIP with SOR, HepG2, and MDA-MB-231were treated with (IC_10_-IC_50_) doses of TQ and PIP, together with SOR (1.0 – 40.0), then incubated for 48 hs before performing the MTT assay as mentioned above.

### Cell line treatment

Cells were treated with half of the predetermined calculated IC_50_ values for all cellular and molecular analyses for each compound. Both cell lines were treated as follows: culture media or 0.1% dimethyl sulfoxide (DMSO), controls; single treatment with either TQ or PIP or SOR; double treatment with TQ + PIP or TQ + SOR or PIP + SOR and finally triple treatment with TQ + PIP + SOR. Treatment was performed 48 hs before the respective analysis, and experiments were repeated at least three times.

### Cell cycle analysis

Cell cycle distribution analysis was carried out by cell cycle assay kit, Elabscience Biotechnology Co., Ltd (Houston, Texas, USA). Following trypsinization, cells were centrifuged at 300 × *g* for 5 min, resuspended using PBS, and 1.2 ml ethanol was added, and the tube was stored at -20 °C for 1 h then, cells were pelleted by centrifugation, the cell pellet was washed with PBS. 100 μl RNase A reagent was added to resuspend the cells and incubated at 37 °C water bath for 30 min then 400 μl propidium iodide (PI) staining solution was added, mixed, and incubated at 4 °C for 30 min. Finally**,** cells were analyzed using proper machine settings.

### Assessment of apoptosis and necrosis by Annexin V-FITC/PI staining

The influence of TQ, PIP, and SOR on apoptosis in HepG2 and MDA-MB-321 cells were quantified by flow cytometry. In brief, cells were collected, washed with PBS, resuspended in 500 μl of annexin V binding buffer, and added 5 μl of annexin V-FITC/PI solution. Cells were resuspended and darkly incubated at 22º C for 20 min before FACS analysis.

### RNA extraction, cDNA synthesis, and quantitative real-time PCR

The mRNA levels of DNA methyltransferase (*DNMT3B*), histone deacetylase (*HDAC3*) genes, and miRNA-29c were assessed by qRT-PCR. Total RNA was first isolated using the miRNeasy Mini Kit (Qiagen, Germany) and reverse‑transcribed to cDNA using the QuantiTect Reverse Transcription kit (Qiagen, Germany). Second, qRT-PCR was performed using the qPCR Master Mix kit (Enzynomics, Korea). The qRT-PCR cycles consisted of 10 min at 95 °C, 40 cycles of denaturation at 95 °C for 10 s, annealing at 60 °C for 15 s, and extension at 72 °C for 15 s. The primers for *DNMT3B*, *HDAC3*, *miRNA-29c*, *β-actin,* and *U6* genes are listed in Table 2. The relative expression of *DNMT3B* and *HDAC3* were calculated by the comparative 2^−ΔΔCt^ method [27] using the endogenous *β-actin* as a housekeeping gene, while *miRNA-29c* was calculated using the *U6* gene as an endogenous control.

**Table 2:** Primers used in real-time PCR amplification

Genes	Primers' sequences
*DNMT3B* forward	5′-TACACAGACGTGTCCAACATGGGC-3′
*DNMT3B* reverse	5′- GGATGCCTTCAGGAATCACACCTC-3′
*HDAC3* forward	5′-ACGTGGGCAACTTCCACTAC-3′
*HDAC3* reverse	5′- GACTCTTGGTGAAGCCTTGC -3′
*β-actin* forward	5′- CGAGCACAGAGCCTCGCCTTTGCC-3′
*β-actin* reverse	5′- TGTCGACGACGAGCGCGGCGATAT -3′
*miRNA-29c* forward	5′- TTT GTC TAG CAC CAT TTG-3′
*miRNA-29c* reverse	5′- CCA GTG CAG GGT CCG AGG TA-3′
*U6* forward	5′- ATTGGAACGATACAGAGAAGATT -3′
*U6* reverse	5′-GGAACGCTTCACGAATTTG-3′

*DNMT3B * DNA methyltransferase, *HDAC3 * Histone deacetylase

### Molecular docking

To perform molecular docking of TQ, PIP, and SOR against DNMT3B (target site PDB ID: 6KDL), HDAC3 (target site PDB ID: 4A69), and vascular endothelial growth factor receptor-2 (VEGFR-2) (target site PDB ID:3V2A), we first downloaded from RCSB PDB database (https://www.rcsb.org/) and prepared by BIOVIA Discovery Studio (Vélizy-Villacoublay, France) [28–30]. The 6KDL retrieved from PDB is the human DNMT3B-DNMT3L complex, where the A and D chains represent DNMT3B. Also, HDAC3 was represented as A and B chains. Therefore, we selected the A chain of 6KDL, 4A69, and 3V2A for protein preparation by removal of water molecules and all ligands in addition to energy minimization and refinement processes.

In addition, the 3D structures of TQ, PIP, and SOR were obtained from the PubChem database (https://pubchem.ncbi.nlm.nih.gov/). The binding free energy, binding affinity (p*Ki*), and the ligand efficiency of TQ, PIP, and SOR against prepared DNMT3B (6KDL-A) and HDAC3 (4A69-A) were determined using InstaDock software [31]. Finally, BIOVIA Discovery Studio Visualizer software did the visualization of target-ligand interaction.

### Statistical analysis

Statistical analysis was performed using GraphPad prism 8.4.2 (https://www.graphpad.com/). Data were represented as mean ± SEM of three independent experiments. One-way ANOVA followed by Tukey’s multiple comparison tests were used to compare group differences. *p* < 0.05 was deemed to show statistical significance.

## Results

### TQ and PIP enhanced the cytotoxicity of SOR in liver and breast cancer cells

**Fig. 1:**
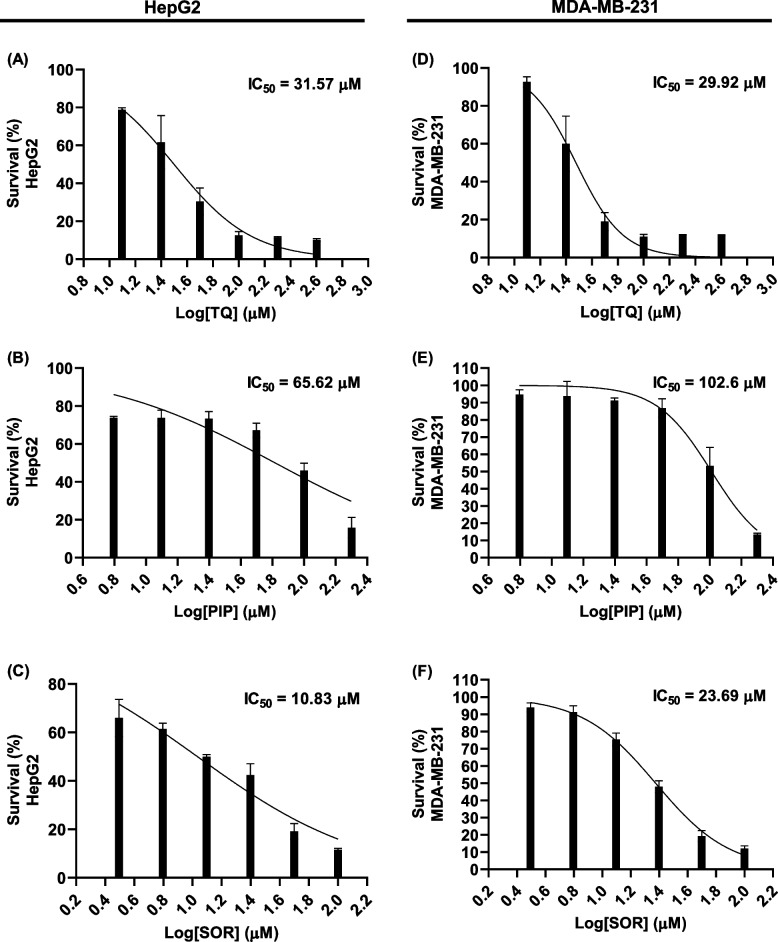
Inhibitory effect of thymoquinone (TQ), piperine (PIP), and sorafenib (SOR) on the proliferation of HepG2 and MDA-MB-231 cells**:** Cells were exposed to different concentrations of each compound for 48 hs and cell viability was determined by MTT assay

The antiproliferative effect of TQ, PIP, and SOR was investigated in HepG2 and MDA-MB-231 cells after 48 hs treatments using the MTT assay. Dose–response curves were used to determine the IC_50_ values for TQ, PIP, and SOR. A dose-dependent growth inhibition was observed in TQ, PIP, and SOR-treated cells compared to control cells. In HepG2 cells, the IC_50_ values for TQ, PIP, and SOR were 31.57, 65.62, and 10.83 µM, respectively. On the other hand, the IC_50_ for the same compounds in MDA-MB-231 cells were 29.92, 102.6, and 23.69 µM, respectively (Figs. 1A-F).

The IC_50_ values for the combination treatments were investigated and presented in Table 3. In HepG2 cells, treatment with different concentrations (IC_10_—IC_50_) of TQ and PIP alone and in combination resulted in a significant decrease in the IC_50_ value for SOR, with the maximum reduction in IC_50_ (85.78%) detected in the combined treatment with the predetermined IC_50_ for TQ and PIP. Similarly, the same combined treatment made the SOR IC50 in MDA-MB-231 cells drop by 75.13%.

**Table 3:** IC_50_ values in (µM) and (%) change for Sorafenib in combination with different doses of Thymoquinone (TQ) and/or Piperine (PIP) against HepG2 and MDA-MB-231

	**IC**_**50**_** of SOR (% change)**	
	**HepG2**	**MDA-MB-231**
**Sorafenib alone**	**10.83 ± 0.54 (0)**	**23.69 ± 0.85 (0)**
**SOR + TQ IC**_**10**_	10.21 ± 0.41 (5.7)	22.24 ± 0.98 (6.1)
**SOR + TQ IC**_**20**_	8.55*** ± 0.24 (21.05)	19.48* ± 0.57 (17.77)
**SOR + TQ IC**_**30**_	5.05*** ± 0.81 (53.3)	15.75*** ± 0.75 (33.51)
**SOR + TQ IC**_**40**_	4.54*** ± 0.32 (58.7)	12.63*** ± 0.35 (46.68)
**SOR + TQ IC**_**50**_	3.91*** ± 0.45 (63.89)	11.75*** ± 0.47 (50.4)
**SOR + PIP IC**_**10**_	10.52 ± 0.72 (2.86)	21.35 ± 0.79 (9.87)
**SOR + PIP IC**_**20**_	10.24 ± 0.47 (5.44)	19.20* ± 0.53 (18.95)
**SOR + PIP IC**_**30**_	7.57** ± 0.82 (30.1)	14.75*** ± 0.437 (37.73)
**SOR + PIP IC**_**40**_	5.12*** ± 0.22 (52.72)	10.85*** ± 0.48 (54.2)
**SOR + PIP IC**_**50**_	3.14*** ± 0.18 (71.0)	10.5*** ± 0.37 (55.67)
**SOR + TQ IC**_**10**_** + PIP IC**_**10**_	9.34* ± 0.42 (13.7)	19.54* ± 0.82 (17.51)
**SOR + TQ IC**_**20**_** + PIP IC**_**20**_	7.88*** ± 0.29 (27.23)	16.33*** ± 0.57 (31.06)
**SOR + TQ IC**_**30**_** + PIP IC**_**30**_	4.24*** ± 0.32 (60.84)	12.47*** ± 0.90 (47.36)
**SOR + TQ IC**_**40**_** + PIP IC**_**40**_	3.17*** ± 0.40 (70.72)	10.48*** ± 0.37 (55.76)
**SOR + TQ IC**_**50**_** + PIP IC**_**50**_	1.54*** ± 0.12 (85.78)	5.89*** ± 0.65 (75.13)

Data are represented as mean ± standard derivation of three independent experiments. Student paired t-test was used to determine significantly-different IC_50_ values (µM) compared to Sorafenib IC_50_; * *p* ˂ 0.05, ** *p* ˂ 0.01, and *** *p* ˂ 0.001

**Fig. 2:**
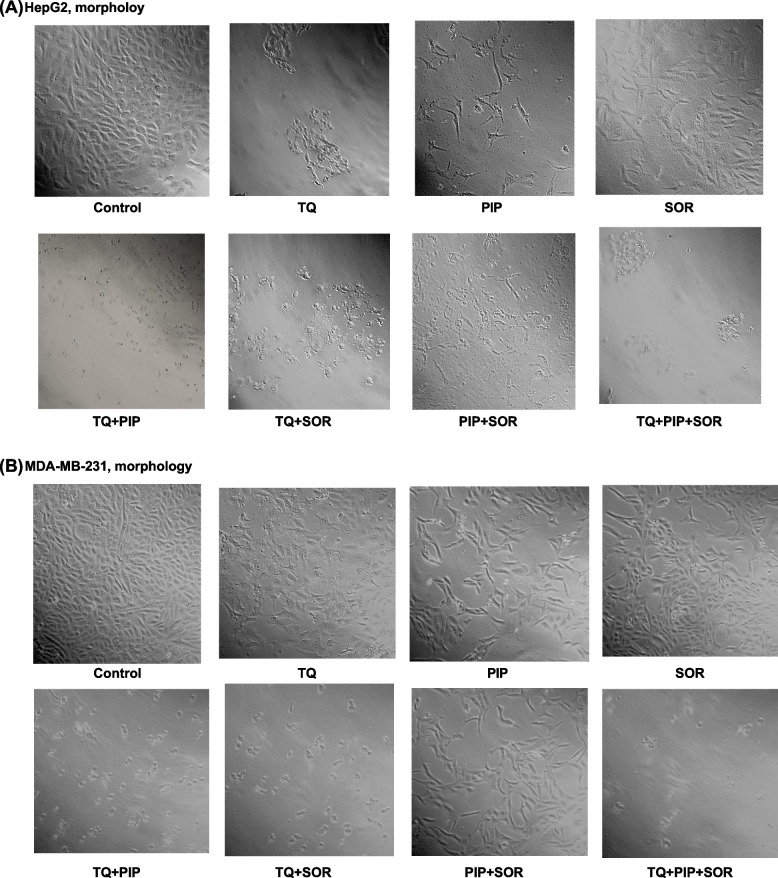
Representative photographs showing morphological changes in HepG2 and MDA-MB-231 cells after treatment with ½ IC_50_s of single or combined thymoquinone (TQ), piperine (PIP), and sorafenib (SOR). Cells were examined and photographed under light microscope 200x

Microscopic examination of cells after exposure to TQ, PIP, and SOR for 48 hs showed various degrees of cytotoxicity depending on the treatment and cell line (Figs. 2A-B). HepG2 and MDA-MB-231 showed noticeable lower cell numbers, reduced viability, and morphological alterations such as losing normal architecture, cell shrinkage appearing as round, wrinkled in shape, condensation of cytoplasm, and increase in membrane roughness and losing the capacity to attach to the culture-plate surface. The highest cytotoxicity was observed after treatment of HepG2 with TQ + PIP + SOR and MDA-MB-231 with TQ + SOR. In contrast, control cells displayed no observable morphological change or death signs and grew efficiently, reaching 100% confluency while adhering to the cell culture plate.

### Determination of cell death mechanism

**Fig. 3:**
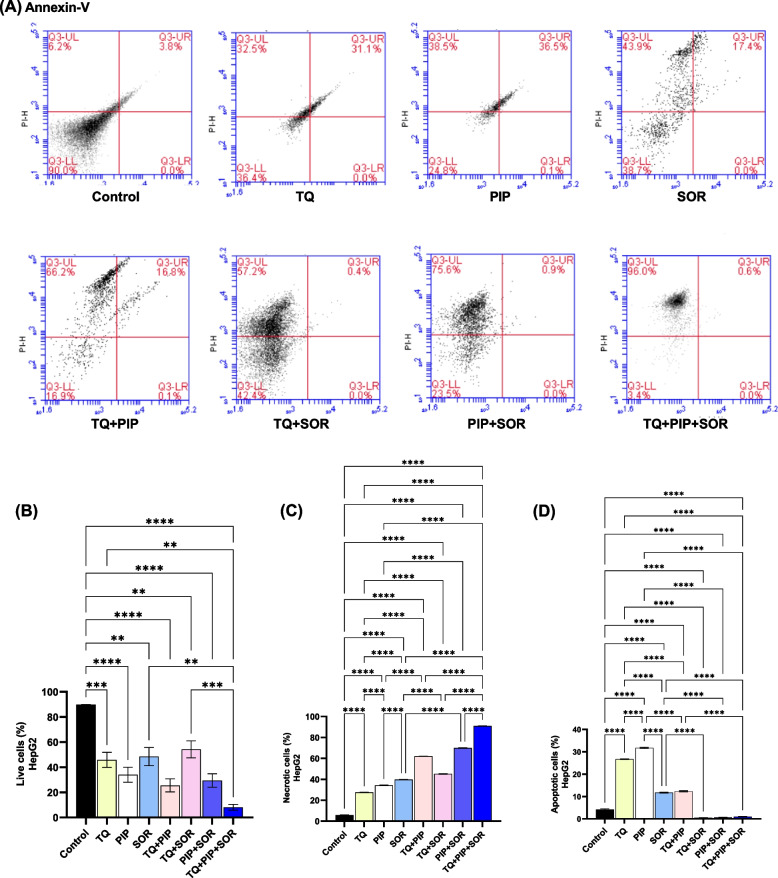
Assessment of cell death mechanism in HepG2 after thymoquinone (TQ), piperine (PIP), and sorafenib (SOR) alone and combination treatments for 48 hs. Cells were double stained with annexin V-FITC and PI, analyzed by flow cytometry. Representative dot plots are shown (**A**). Percentage of live cells, necrotic cells and apoptotic (early + late) (**B**, **C** and **D**). Cell populations were plotted and represented collectively as percentage of total events. Data are expressed as the mean ± SEM; *n* = 3. Means within columns carrying * are significantly different at (*p* ˂0.05), ** (*p* ˂ 0.01), *** (*p* ˂ 0.001) and **** (*p* ˂ 0.0001)

**Fig. 4:**
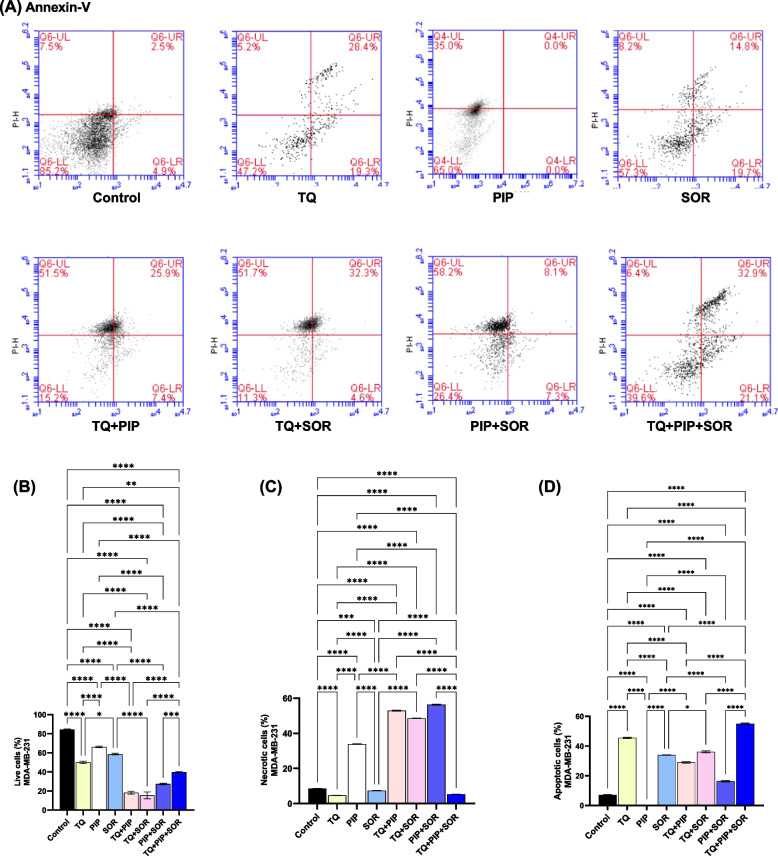
Assessment of cell death mechanism in MDA-MB-231 after thymoquinone (TQ), piperine (PIP), and sorafenib (SOR) alone and combination treatments for 48 hs. Cells were double stained with annexin V-FITC and PI, analyzed by flow cytometry. Representative dot plots are shown (**A**), percentage of live cells, necrotic cells and apoptotic (early + late) (**B**, **C** and **D**). Cell populations were plotted and represented collectively as percentage of total events. Data are expressed as the mean ± SEM; *n* = 3. Means within columns carrying * are significantly different at (*p* ˂0.05), ** (*p* ˂ 0.01), *** (*p* ˂ 0.001) and **** (*p* ˂ 0.0001)

The percentages of viable and dead cells were determined after 48 hs of treatment by double annexin V-FITC/PI staining (Figs. 3A and 4A). Flow cytometry analysis indicated that the highest cytotoxic effect showing the lowest number of viable cells was observed after treatment of HepG2 with TQ + PIP + SOR (8.10% ± 2.37) and MDA-MB-231 with TQ + SOR (15.33% ± 3.59) (Figs. 3B and 4B).

The cell death mechanism (apoptosis vs. necrosis) was investigated using flow cytometry by analyzing the cells in (early + late) apoptosis vs. necrotic cells. The highest percentage of apoptotic cells were detected after treatment of HepG2 with PIP (31.77% ± 0.29) and MDA-MB-231 cells with TQ + PIP + SOR (55.0% ± 0.46) (Figs. 3D and 4D).

On the other hand, the highest percentage of necrosis was observed after treatment of HepG2 with TQ + PIP + SOR (90.97% ± 0.03) and MDA-MB-231 with PIP + SOR (56.33% ± 0.24) (Figs. 3C and 4C).

### Effect of TQ, PIP, and SOR on cell cycle phases

**Fig. 5:**
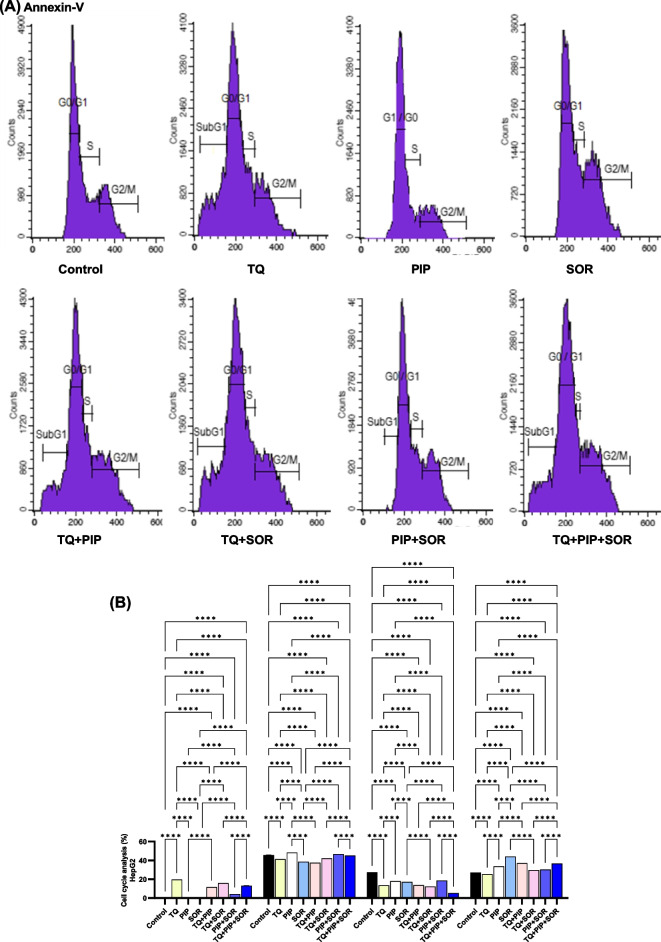
Effect of thymoquinone (TQ), piperine (PIP), and sorafenib (SOR) and their combinations on cell cycle distribution of HepG2 cells. Cell cycle analysis was performed using flow cytometry. Representative histograms are shown (**A**), Percentage of cells in each cell cycle phases (**B**). Data are expressed as the mean ± SEM; *n* = 3. Means within columns carrying * are significantly different at (*p* ˂0.05), ** (*p* ˂ 0.01), *** (*p* ˂ 0.001) and **** (*p* ˂ 0.0001)

**Fig. 6:**
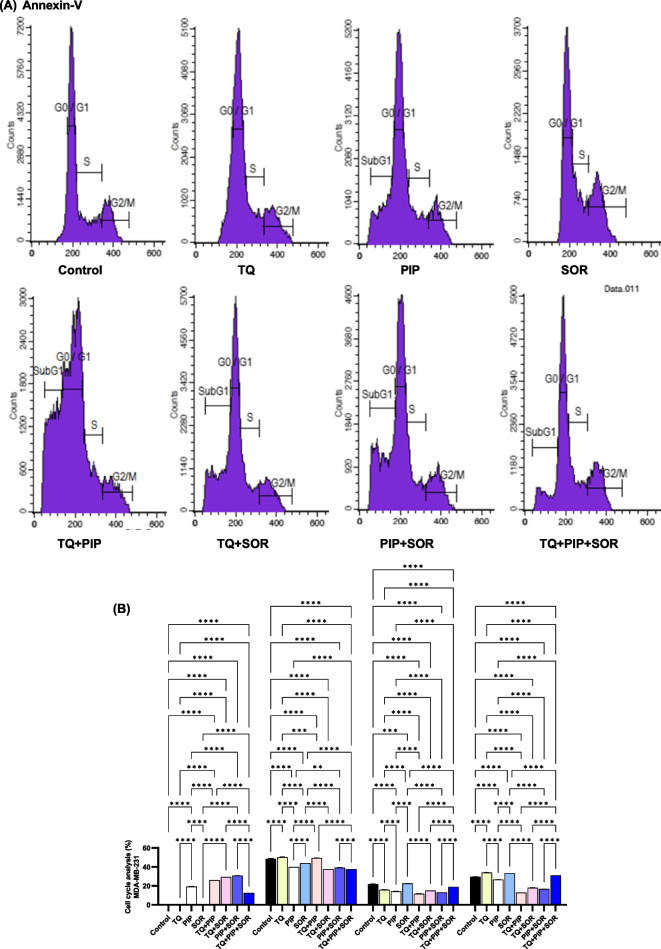
Effect of thymoquinone (TQ), piperine (PIP), and sorafenib (SOR) and their combinations on cell cycle distribution of MDA-MB-231 cells. Cell cycle analysis was performed using flow cytometry. Representative histograms are shown (**A**), Percentage of cells in each cell cycle phases (**B**). Data are expressed as the mean ± SEM; *n* = 3. Means within columns carrying * are significantly different at (*p* ˂0.05), ** (*p* ˂ 0.01), *** (*p* ˂ 0.001) and **** (*p* ˂ 0.0001)

The effects of TQ, PIP, and SOR and their combination on the HepG2 and MDA-MB-231 cell cycle progression were evaluated by assessment of DNA content using flow cytometry. Representative histogram data files and cell cycle phases plotted as the percentage of total gated events are shown in Figs. 5A and 6A. In HepG2, all TQ, PIP, SOR, and their combinations treatments caused a significant G2/M cell cycle arrest, with SOR showing the highest (44.16% ± 0.1, *p* < 0.0001) significant arrest compared to control cells (27.01% ± 0.5) (Fig. 5B). Simultaneously, all treatments significantly reduced the cell percentage undergoing DNA synthesis compared to control cells. The highest significant subG1 peak representing cells with low fragmented DNA was detected in TQ-treated cells (19.61% ± 0.7, *p* < 0.0001) (Fig. 5B). On the other hand, in MDA-MB-231, TQ followed by SOR single treatments caused the highest significant arrest and cell accumulation in the G2/M (33.93% ± 0.10) and (33.45% ± 0.03), respectively (Fig. 6B). Finally, the highest significant subG1 cell population was detected in PIP + SOR treated cells (30.81% ± 0.5, *p* < 0.0001), (Fig. 6B).

### TQ, PIP, and SOR reduced DNMT3B/HDAC3 and increased miRNA-29c expression

The effect of TQ, PIP, and SOR treatment on the expression of *DNMT3B*/*HDAC3* genes and the tumor suppressor miRNA-29c was examined by RT- PCR.

**Fig. 7:**
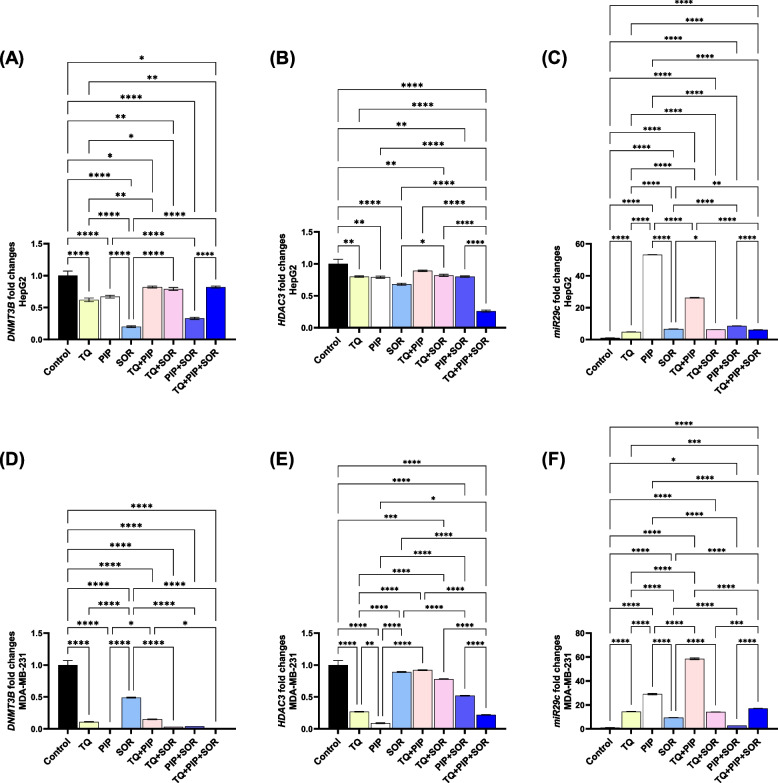
Real time-PCR analysis for epigenetic-related genes [DNA methyltransferase (DNMT3B), histone deacetylase (HDAC3) and miRNA-29c] after thymoquinone (TQ), piperine (PIP), and sorafenib (SOR) treatments for 48 hs in HepG2 (**A**-**C**) and MDA-MB-231 (**D**-**F**) cells. The data provided are mean ± SEM (*n* = 3). Means within columns carrying * are significantly different at (*p* ˂0.05), ** (*p* ˂ 0.01), *** (*p* ˂ 0.001) and **** (*p* ˂ 0.0001)

*DNMT3B* expression analysis represented in Fig. 7A showed that in HepG2 cells, SOR treatment caused the highest reduction in expression (approximately 5 folds) compared to control. On the other hand, PIP and TQ + PIP + SOR treatments suppressed *DNMT3B* expression almost entirely in MDA-MB-231 cells (Fig. 7D).

The highest decrease in *HDAC3* expression was detected after combined TQ + PIP + SOR (3.8-fold) in HepG2 cells and PIP single treatment (11-fold) in MDA-MB-231, (Figs. 7B and 7E).

Finally, PIP alone and combined with TQ caused the most significant increase in miRNA-29c expression in HepG2 (53-fold) and MDA-MB-231 (58 folds), respectively, compared to control cells (Figs. 7C and 7F).

### Molecular Docking Study

**Fig. 8:**
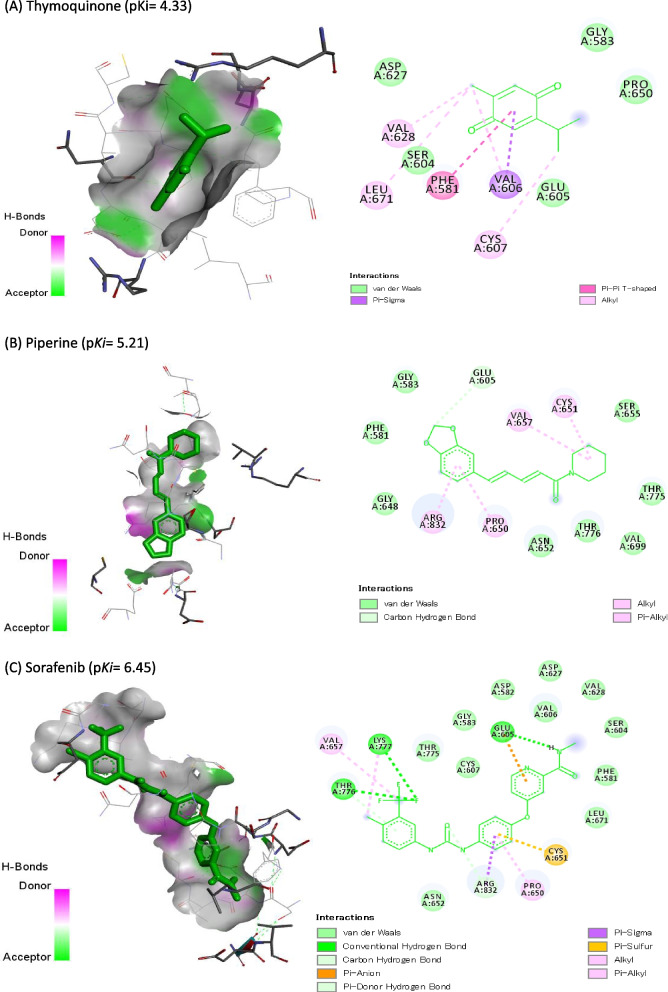
Co-crystallized ligands redocked in DNA methyltransferase (DNMT3B), hydrogen bonds (green) and the pi interactions are represented in purple lines with mapping surface showing thymoquinone (TQ), piperine (PIP), and sorafenib (SOR) occupying the active pocket of DNMT3B

**Fig. 9:**
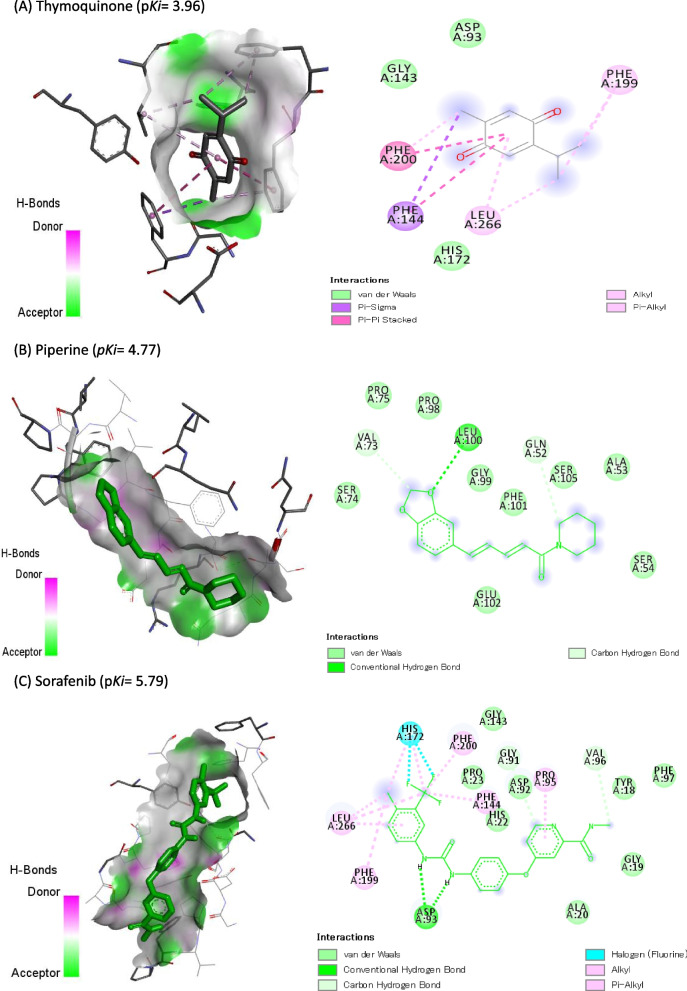
Co-crystallized ligands redocked in histone deacetylase (HDAC3), hydrogen bonds (green) and the pi interactions are represented in purple lines with mapping surface showing thymoquinone (TQ), piperine (PIP), and sorafenib (SOR) occupying the active pocket of HDAC

**Fig. 10:**
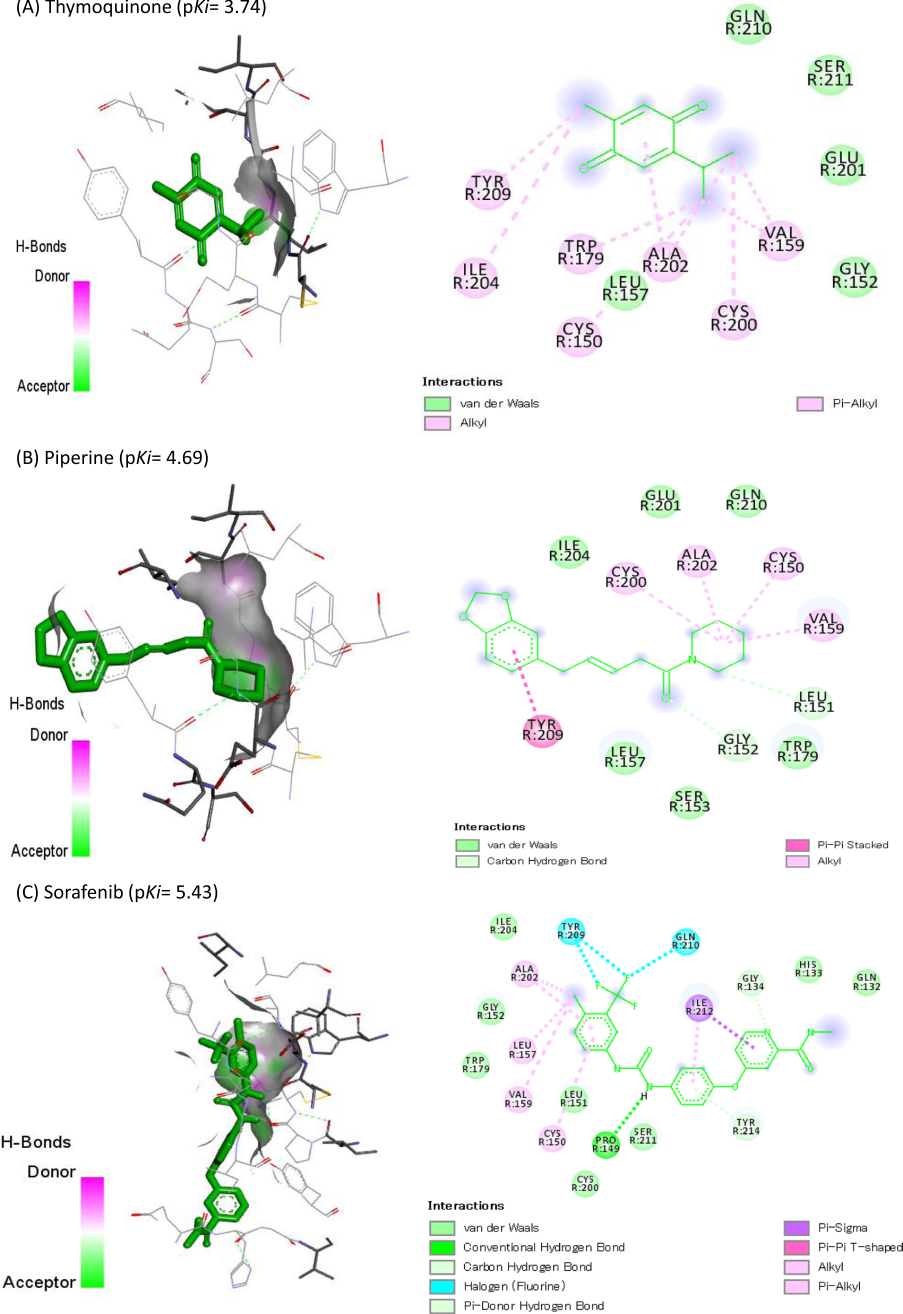
Co-crystallized ligands redocked in vascular endothelial growth factor receptor-2 (VEGFR-2), hydrogen bonds (green) and the pi interactions are represented in purple lines with mapping surface showing thymoquinone (TQ), piperine (PIP), and sorafenib (SOR) occupying the active pocket of VEGFR-2

Molecular docking of TQ, PIP, and SOR against DNMT3B (6KDL-A), HDAC3 (4A69-A), and VEGFR-2 (3V2A-A) are represented in Table 4. SOR showed the highest binding affinity (p*Ki*: 6.45), (p*Ki*: 5.79), and (p*Ki*: 5.43) toward DNMT3B, HDAC3, and VEGFR-2, respectively. Also, PIP exhibited p*Ki* of 5.21, 4.77, and 4.69 toward DNMT3B, HDAC3, and VEGFR-2, respectively, while TQ exhibited the lowest affinities (4.33, 3.96, and 3.74, respectively). Co-crystallized ligands redocked in DNMT3B, HDAC3, and VEGFR-2 are shown in Figs. 8, 9, and 10, respectively.

**Table 4:** Docking score of thymoquinone (TQ), piperine (PIP), and sorafenib (SOR) against DNA methyltransferase (DNMT3B) (target site PDB ID: 6KDL-A), histone deacetylase (HDAC3) (target site PDB ID: 4A69-A), and vascular endothelial growth factor receptor-2 (VEGFR-2) (target site PDB ID:3V2A-A)

Name of the ligand	DNMT3B (target site PDB ID: 6KDL-A)			HDAC3(target site PDB ID: 4A69-A)			VEGFR-2 (target site PDB ID:3V2A-A)		
	Binding Free Energy (kcal/mol)	p*Ki*	Ligand Efficiency (kcal/mol/non-H atom)	Binding Free Energy (kcal/mol)	p*Ki*	Ligand Efficiency (kcal/mol/non-H atom)	Binding Free Energy (kcal/mol)	p*Ki*	Ligand Efficiency (kcal/mol/non-H atom)
**Thymoquinone (TQ)**	-5.9	4.33	0.4917	-5.4	3.96	0.45	-5.1	3.74	0.425
**Piperine (PIP)**	-7.1	5.21	0.3381	-6.5	4.77	0.3095	-6.4	4.69	0.3048
**Sorafenib (SOR)**	-8.8	6.45	0.275	-7.9	5.79	0.2469	-7.4	5.43	0.2313

TQ, PIP, and SOR interacted with the amino acid residues in the binding site of DNMT3B by 6, 3, and 11 bonds, respectively (Table 5). Hydrophobic interactions were recognized between TQ (1 with PHE:581, 1 with VAL606, 1 with PRO650, and 2 with ARG832), PIP (1 with PRO650, 1 with THY776, and 1 with ARG832), and SOR (1 with VAL606, 2 with PRO650, 2 with VAL657, and 1 with ARG832) and DNMT3B binding site. Also, hydrogen bonds were reported between TQ and SER610 and three bonds between SOR and THY776. Furthermore, SOR interacted with three halogen bonds with THY776 and one charge bond with ARG832.

**Table 5:** Molecular interaction of thymoquinone (TQ), piperine (PIP), and sorafenib (SOR) against DNA methyltransferase (DNMT3B) (target site PDB ID: 6KDL-A)

		**Thymoquinone (TQ)**	**Piperine (PIP)**	**Sorafenib (SOR)**
**Total**	Favorable	6	3	11
	Charge	0	0	1
	Halogen	0	0	3
	Hydrophobic	5	3	6
	Hydrogen	1	0	3
	Other	0	0	1
**Favorable**	A: PHE591	1	0	0
	A: VAL606	1	0	1
	A: SER610	1	0	0
	A: PRO650	1	1	2
	A: CYS651	0	0	1
	A: VAL657	0	0	2
	A: THR776	0	1	3
	A: ARG832	2	1	2
**Charge**	A: ARG832	0	0	1
**Halogen**	A: THR776	0	0	3
**Hydrophobic**	A: PHE581	1	0	0
	A: VAL606	1	0	1
	A: PRO650	1	1	2
	A: VAL657	0	0	2
	A: THR776	0	1	0
	A: ARG832	2	1	1
**Hydrogen**	A: SER610	1	0	0
	A: THR776	0	0	3
**Other**	A: CYS651	0	0	1

Eight hydrophobic interactions were recognized between TQ and PHE144 (2), PHE199 (2), PHE200 (2), and LEU266 (2) in HDAC3’s binding site (Table 6). Also, SOR made nine hydrophobic interactions with PRO095 (1), PHE144 (1), HIS172 (2), PHE199 (1), PHE200 (1), and LEU266 (3). In addition, SOR is bound with LEU233 by three halogen bonding, while bound with GLY91 (1), ASP93 (2), and VAL96 (1) with hydrogen bonds. PIP exhibited only three hydrogen bonds with GLU52, VAL73, and LEU100.

**Table 6:** Molecular interaction of thymoquinone (TQ), piperine (PIP), and sorafenib (SOR) against histone deacetylase (HDAC3) (target site PDB ID: 4A69-A)

		**Thymoquinone (TQ)**	**Piperine (PIP)**	**Sorafenib (SOR)**
**Total**	Favorable	8	3	15
	Halogen	0	0	2
	Hydrophobic	8	0	9
	Hydrogen	0	3	4
**Favorable**	A: GLN52	0	1	0
	A: VAL73	0	1	0
	A: GLY91	0	0	1
	A: ASP93	0	0	2
	A: PRO95	0	0	1
	A: VAL96	0	0	1
	A: LEU100	0	1	0
	A: PHE144	2	0	1
	A: HIS172	0	0	4
	A: PHE199	2	0	1
	A: PHE200	2	0	1
	A: LEU233	2	0	3
**Halogen**	A: HIS172	0	0	2
**Hydrophobic**	A: PRO95	0	0	1
	A: PHE144	2	0	1
	A: HIS172	0	0	2
	A: PHE199	2	0	1
	A: PHE200	2	0	1
	A: LEU266	2	0	3
**Hydrogen**	A: GLN52	0	1	0
	A: VAL73	0	1	0
	A: GLY91	0	0	1
	A:ASP93	0	0	2
	A: VAL96	0	0	1
	A: LEU100	0	1	0

The molecular interaction between TQ, PIP, and SOR with VEGFR-2 is represented in Table 7, where they are bound by 4, 3, and 10 bonds, respectively with VEGFR-2’s binding site. TQ bound with PHE258 (2) and PHE288 (1) with three hydrophobic interactions, while PIP bound with VAL171 by one hydrophobic bond. SOR made five hydrophobic interactions with CYC150 (1), VAL159 (1), GLN210 (1), ILE212 (1), and TYR214 (1), while bound with two halogen bindings with GLY134 and TYR214.

**Table 7:** Molecular interaction of thymoquinone (TQ), piperine (PIP), and sorafenib (SOR) against vascular endothelial growth factor receptor-2 (VEGFR-2) (target site PDB ID:3V2A-A)

		**Thymoquinone (TQ)**	**Piperine (PIP)**	**Sorafenib (SOR)**
**Total**	Favorable	4	3	10
	Halogen	0	0	2
	Hydrophobic	3	2	5
	Hydrogen	1	1	5
**Favorable**	A: GLY134	0	0	1
	A: CYS150	0	0	1
	A: GLY152	0	0	2
	A: ASN158	0	2	0
	A: VAL159	0	0	1
	A: VAL171	0	1	0
	A: GLN210	0	0	1
	A: SER211	0	0	1
	A: ILE212	0	0	1
	A: TYR214	0	0	2
	A: PHE258	2	0	0
	A: ARG275	1	0	0
	A: PHE288	1	0	0
**Halogen**	A: GLY134	0	0	1
	A: TYR214	0	0	1
**Hydrophobic**	A: CYC150	0	0	1
	A: ASN158	0	1	0
	A: VAL159	0	0	1
	A: VAL171	0	1	0
	A: GLN210	0	0	1
	A: ILE212	0	0	1
	A: TYR214	0	0	1
	A: PHE258	2	0	0
	A: PHE288	1	0	0
**Hydrogen**	A: GLY134	0	0	1
	A: GLY152	0	0	2
	A: ASN158	0	1	0
	A: SER211	0	0	1
	A: TYR214	0	0	1
	A: ARG275	1	0	0

## Discussion

SOR was the first systemic compound prescribed by US FDA that significantly increased the survival rate for liver cancer patients [32]. However, the one major disadvantage of SOR is its high toxicity, especially at high doses [33]. Therefore, testing new natural agents that might enhance its effects and allow lower doses, would potentially minimize this toxicity [34]. TQ and PIP are two natural compounds commonly used for medicinal purposes [35, 36]. To the best of our knowledge, combining TQ or PIP with SOR has not been investigated. One study on breast carcinoma xenograft reported a combination between TQ and PIP [37].

In the current study, we showed that combinations of TQ and/or PIP with SOR have significantly enhanced the latter anti-proliferative and cytotoxic effects in both HepG2 and MDA-MB231 cells with variable potency. Overall, HepG2 cells were more responsive to PIP or SOR since both compounds showed lower IC_50_ values compared with MDA-MB231 cells. This finding agrees with the fact that SOR is prescribed mainly in the treatment of hepatocellular carcinomas [38]. Interestingly, the IC50 values for SOR were significantly diminished in both HepG2 and MDA-MB-231 cells (up to 85% and 75%, respectively) after combined treatment with TQ and/or PIP at different doses. As a consequence, to reduce clinical doses and reduce the common side effects associated with high doses of chemotherapeutic drugs, it is crucial to decrease the 50% inhibitory cytotoxic dose of the chemotherapeutic drug SOR while maintaining its overall cytotoxicity. This could be done through the actions of the natural compounds TQ and PIP.

TQ and PIP alone treatments showed anti-proliferative effects, which agrees with previous studies that reported a decrease in viability of lymphocyte leukaemia cells when treated with TQ [39]. Another study showed that TQ reduced the cell viability of the Huh-7 hepatocellular carcinoma cell line in a dose-dependent manner [40]. On the other hand, Greenshields et al. observed that PIP reduced the proliferation and invasion of MDA-MB-231 cells [41].

Many recent reports, including the current study, indicated promising chemo-modulatory actions of TQ when combined with other chemotherapeutic agents used against various cancers [42, 43]. In 2020, it was reported that TQ enhanced docetaxel efficiency in MDA-MB-231 and MCF-7 cells by reducing its effective dose [44]. Moreover, TQ synergistically improved the anticancer activity of doxorubicin and cisplatin in hepatocellular carcinoma HepG2 cells [45]. Similarly, PIP was also reported to exert chemo-modulatory effects on chemotherapeutic drugs in different cancers [46, 47].

We have investigated the potential mechanisms causing this anti-proliferative effect, such as modulation of cell cycle interphase, cell death (apoptosis vs. necrosis), and epigenetic genome modifications. The cell cycle is vital in maintaining cell growth and tissue homeostasis, abnormalities in cell cycle progression results in serious diseases, including cancer [48]. We showed that SOR single treatment has resulted in significant G2/M cell cycle arrest in HepG2 and MDA-MB231 cells, which agrees with a previous study that was performed on Hep3B, HepG2, PLC-PRF-5, and SK-Hep1, human hepatocellular carcinoma cell lines [49]. However, other studies reported SOR to arrest cells at G0/G1 phase [50, 51]. Similar to SOR and TQ caused a significant G2/M arrest, TQ was previously reported to cause a G2/M phase arrest in HepG2 cells [52]. However, others have reported that exposure of HepG2 cells to TQ arrested cells at the G0/G1 phase [53]. Depending on the cell line under investigation and tumor behaviour, PIP and TQ were previously suggested to cause cell cycle arrest at G0/G1 and G2/M phases by binding to different cell cycle regulatory proteins [54, 55]. This observation explains the different cell cycle arrest phases that might be observed using the same compound.

Impaired apoptosis is reported as one of the mechanisms leading to SOR resistance in cancer cells and the antiapoptotic protein, B-cell lymphoma 2, is suggested to modulate this phenomenon [50]. Many reports have attributed the anticancer effects of TQ and PIP to its selective proapoptotic actions through the regulation of p53, Bcl-2-associated X protein, and B-cell lymphoma 2 equilibrium and activation of the caspase enzymes [46, 56]. We have examined the apoptotic and necrotic cell death after individual and combined TQ, PIP, and SOR treatment. Notably, necrosis-related cell death was the most observed cause of cell death in HepG2 cells regardless of the treatment. In contrast, in MDA-MB231 cells, apoptotic cell death was prevalent in TQ and SOR single treatments and triple TQ + PIP + SOR treatments.

Interestingly, a combination of TQ or PIP with SOR increased the population of necrotic cells by 6.5 and 8 folds, respectively, compared to SOR alone treatment. Such data might be important in future combination therapy studies aiming to bypass apoptosis as a major cell death mechanism in SOR-resistant cancer cells. Bypassing cancer drug resistance by induction of necroptosis is under clinical investigation as a promising therapeutic mechanism against apoptosis-resistant cancer cells [57].

The current study showed that both TQ and PIP potentiated the anti-*HDAC3* and anti-*DNMT3B* effect of SOR, causing a significant drop in *HDAC3* and *DNMT3B* expression levels. HDACs and *DNMT3B* act as transcriptional repressors for tumor suppressor genes, high mRNA levels of *HDAC3* and *DNMT3B* are associated with poor prognosis in patients with different types of cancer [58, 59]. Combination therapy with histone deacetylases (HDACi) or (*DNMT3B*) inhibitors are currently under investigation for achieving a full cancer therapeutic potential [60, 61]. Several studies have already reported the synergistic effects of treatment with HDACi and various chemotherapeutic drugs [62, 63]. SOR, TQ, and PIP were reported to act as HDACi and DNMT3B inhibitors [64–66]. Furthermore, molecular docking revealed that TQ, PIP, and SOR formed hydrophobic and hydrogen bonds with DNMT3B and HDACi, indicating their affinity for inhibiting DNMT3B and HDACi.

In the same way, TQ, PIP, and SOR can bind effectively to VEGFR-2 and inhibit angiogenesis. More research is needed to figure out how TQ, PIP, SOR, and their combinations affect cancer angiogenesis.

The miR-29C has been identified as a crucial miRNA in several cancers; it regulates several oncogenic processes, including epigenetics. The miR-29 family was reported to function as tumor suppressor microRNA through sponging DNMT3A and DNMT3B. However, the biological activity of miR-29C in cancer development and progression is still disputed, most studies reported miR-29C as a tumor suppressor, and others suggested it acts as an oncogene [67]. miR-29b levels were oppositely related to HDAC levels in vitro and human tissue [68]. To the best of our knowledge, the effect of SOR, TQ, and PIP on miR-29C expression levels was not investigated before; the current study confirmed the tumor suppressor role of miR-29C since all our treatments have caused a significant increase in miR-29C expression levels with the highest increase detected in PIP alone (53-fold in HepG2) and PIP + TQ (58-fold in MDA-MB-231).

The molecular docking study was based on assessing the binding energy and binding affinity of ligand-receptor interactions as reflected by the docking score in kcal/mol. A lower docking score indicates a higher binding affinity. The molecular docking results showed a strong affinity of SOR to DNMT3B, and HDAC3, followed by PIP and then TQ.

DNA methylation occurs almost exclusively in CpG islands in mammals catalyzed by DNA methyltransferases (DNMTs). DNMT1 has been shown to maintain methylation in somatic cells, and DNMT3a and *DNMT3B* are thought to be involved in de novo DNA methylation in embryonic stem cells and early embryos. It was recently found that DNMT1, DNMT3a, and *DNMT3B* are overexpressed in several human tumors, compared to levels in corresponding normal tissues [69–71]. Preclinical and clinical studies demonstrated that the anticancer properties of bioactive components (i.e., parthenolide, folate, retinoids, etc.) may be attributed to its influence on epigenetic processes through binding to DNMT1 enzymatic centre or/and disrupting *DNMT* transcription [72]. Inhibiting *DNMT3B* with RNA interference or a selective *DNMT3B* inhibitor effectively suppressed the DNMT3B activity in vitro and in vivo. Our docking study showed strong binding between SOR, PIP, and TQ to DNMT3B, which inhibited DNMT3B activity. This finding agrees with another study that showed that DNMT3B inhibitor showed a synergistic effect with SOR in the SOR-resistant Hep3B cells [73].

Genome acetylation is one of the most important epigenetic modifications in promoters of genes that regulate the cell cycle, differentiation, cell growth, and survival in cancer [74]. The acetylation of active genes is controlled by the expression and activity of HDAC [75]. Recently, several reports have shown some dietary phytochemicals as HDAC inhibitors [76–78]. HDAC inhibitors lead to increased histone acetylation and transcriptional upregulation of gene expression, inducing cancer cell cycle arrest, apoptosis, and necrosis in a variety of transformed cell lines by several mechanisms depending on the cancer type; HDAC inhibitors, and doses [79]. We showed strong binding between SOR, PIP, and TQ to HDAC, which would probably inhibit HDAC biological activity through disrupting protein–protein interactions important for HDAC activity, as recently suggested [80]. Induction of apoptosis seems to be the predominant route of HDACi-induced cell death [81]; this agrees with our data showing apoptosis as the main cell death mechanism in PIP-treated HepG2 cells.

## Conclusion

The present study showed that TQ and PIP enhance SOR's anti-tumor and cytotoxic effects against hepatocellular carcinoma and triple-negative breast cancer cells. In our study, we studied cell cycle arrest, cell toxicity, apoptosis, and necrosis induction, as well as molecular epigenetic mechanisms that reduce DNMT3B and HDAC3 expression and increase miR-29C expression. Additionally, the results of the molecular docking study validated and supported our in vitro findings.

## Supplementary Information

**Additional file 1. **Sorafenib anticancer potentials.

**Additional file 2. **Cancer clinical trials of sorafenib.

**Additional file 3. **The anticancer potential of Thymoquinone.

**Additional file 4. **Thymoquinone anticancer clinical trials.

**Additional file 5. **Piperine anticancer potentials.

**Additional file 6. **Piperine anticancer clinical trials.

## Data Availability

All data generated or analyzed during this study are included in this published article (and its supplementary information files).

## Abbreviations

DNMT3B: DNA methyltransferase
HDAC3: Histone deacetylase
PIP: Piperine
SOR: Sorafenib
TQ: Thymoquinone

## References

[1] Organization, W.H., *World health statistics 2018: monitoring health for the SDGs, sustainable development goals*. 2018: World Health Organization.

[2] Petrick JL (2020). International trends in hepatocellular carcinoma incidence, 1978–2012. Int J Cancer.

[3] Sung H (2021). *Global cancer statistics 2020: GLOBOCAN estimates of incidence and mortality worldwide for 36 cancers in 185 countries*. CA Cancer J Clin.

[4] Brighton, D. and M. Wood, *The Royal Marsden Hospital handbook of cancer chemotherapy: a guide for the multidisciplinary team*. 2005: Churchill Livingstone.

[5] Kelland, L.R., *Cancer cell biology, drug action and resistance.* The royal marsden hospital handbook of cancer chemotherapy, 2005: p. 1–15.

[6] Scurr, M., I. Judson, and T. Root, *Combination chemotherapy and chemotherapy principles.* Cancer chemotherapy’, Eds Brighton D, Wood M, London: Churchill Livingstone, 2005: p. 17–30.

[7] Thackery, E., *The Gale Encyclopedia of Cancer: LZ*. Vol. 2. 2002: Gale Cengage.

[8] Siegel AB (2010). Sorafenib: where do we go from here?. Hepatology.

[9] Cragg GM, Newman DJ (2005). Plants as a source of anti-cancer agents. J Ethnopharmacol.

[10] Khan H (2014). Medicinal plants in light of history: recognized therapeutic modality. J Evid Based Complementary  Altern Med.

[11] Cortez-Trejo M. C (2022). Potential Anticancer Activity of Pomegranate (Punica granatum L.) Fruits of Different Color: In Vitro and In Silico Evidence. Biomolecules.

[12] Malaluan I. N (2022). Antituberculosis and Antiproliferative Activities of the Extracts and Tetrahydrobisbenzylisoquinoline Alkaloids from Phaeanthus ophthalmicus: In Vitro and In Silico Investigations. Philipp. J. Sci.

[13] Ma L (2021). Plant natural products: promising resources for cancer chemoprevention. Molecules.

[14] Rayan A, Raiyn J, Falah M (2017). Nature is the best source of anticancer drugs: Indexing natural products for their anticancer bioactivity. PLoS ONE.

[15] Imran M (2018). Thymoquinone: A novel strategy to combat cancer: A review. Biomed Pharmacother.

[16] H El-Far, A., *Thymoquinone anticancer discovery: possible mechanisms.* Current drug discovery technologies, 2015. 12(2): p. 80–89. 10.2174/1570163812666150716111821 26264075

[17] El-Far AH, Darwish NH, Mousa SA (2020). Senescent colon and breast cancer cells induced by doxorubicin exhibit enhanced sensitivity to curcumin, caffeine, and thymoquinone. Integr Cancer Ther.

[18] El-Far AH (2021). Thymoquinone and costunolide induce apoptosis of both proliferative and doxorubicin-induced-senescent colon and breast cancer cells. Integr Cancer Ther.

[19] El-Far AH (2022). Curcumin and Thymoquinone Combination Attenuates Breast Cancer Cell Lines’ Progression. Integr Cancer Ther.

[20] El-Far AH (2021). Thymoquinone and its nanoformulation attenuate colorectal and breast cancers and alleviate doxorubicin-induced cardiotoxicity. Nanomedicine.

[21] Saddiq, A.A., et al., *Curcumin, thymoquinone, and 3, 3′-diindolylmethane combinations attenuate lung and liver cancers progression.* Frontiers in Pharmacology, 2022: p. 2563. 10.3389/fphar.2022.936996 PMC9277483 35847018

[22] (USDA Plants Database (2022). Nigella sativa L. black cumin. Available at: https://plants.sc.egov.usda.gov/home/plantProfile?symbol=NISA2. Accessed July 28.

[23] Selvendiran K, Sakthisekaran D (2004). Chemopreventive effect of piperine on modulating lipid peroxidation and membrane bound enzymes in benzo (a) pyrene induced lung carcinogenesis. Biomed Pharmacother.

[24] (USDA Plants Database (2022). Piper nigrum. Available at: https://plants.sc.egov.usda.gov/home/plantProfile?symbol=PINI3. Accessed July 28.

[25] (USDA Plants Database (2022). Piper longum. Available at: https://plants.sc.egov.usda.gov/home/plantProfile?symbol=PILO4. Accessed July 28.

[26] Ismail N (2018). Novel combination of thymoquinone and resveratrol enhances anticancer effect on hepatocellular carcinoma cell line. Future J Pharm Sci.

[27] Livak K.J, Schmittgen T.D (2001). Analysis of relative gene expression data using real-time quantitative PCR and the 2− ΔΔCT method. methods.

[28] Magpantay HD (2021). Antibacterial and COX-2 inhibitory tetrahydrobisbenzylisoquinoline alkaloids from the Philippine medicinal plant Phaeanthus ophthalmicus. Plants.

[29] in silico study (2021). Jayanti, D. A. P. I. S., Abimanyu, I. G. A. M. & Azzamudin, H. Spirulina platensis’s phycocyanobilin as an antiangiogenesis by inhibiting VEGFR2-VEGFA pathway in breast cancer. JSMARTech J Smart Bioprospecting Technol.

[30] Teixeira IS (2022). Computer Modeling Explains the Structural Reasons for the Difference in Reactivity of Amine Transaminases Regarding Prochiral Methylketones. Int J Mol Sci.

[31] Hassan NM (2017). Protein-ligand blind docking using QuickVina-W with inter-process spatio-temporal integration. Sci Rep.

[32] Llovet JM (2008). Sorafenib in advanced hepatocellular carcinoma. N Engl J Med.

[33] Almhanna K, Philip PA (2009). Safety and efficacy of sorafenib in the treatment of hepatocellular carcinoma. Onco Targets Ther.

[34] Morisaki T (2013). Combining celecoxib with sorafenib synergistically inhibits hepatocellular carcinoma cells in vitro. Anticancer Res.

[35] Khader M, Eckl PM (2014). Thymoquinone: an emerging natural drug with a wide range of medical applications. Iran J Basic Med Sci.

[36] Shityakov S (2019). Phytochemical and pharmacological attributes of piperine: A bioactive ingredient of black pepper. Eur J Med Chem.

[37] Talib WH (2017). Regressions of breast carcinoma syngraft following treatment with piperine in combination with thymoquinone. Sci Pharm.

[38] Raoul J-L (2019). Sorafenib: experience and better management of side effects improve overall survival in hepatocellular carcinoma patients: a real-life retrospective analysis. Liver Cancer.

[39] Salim LZA (2013). Thymoquinone induces mitochondria-mediated apoptosis in acute lymphoblastic leukaemia in vitro. Molecules.

[40] Ashour AE (2014). Thymoquinone suppression of the human hepatocellular carcinoma cell growth involves inhibition of IL-8 expression, elevated levels of TRAIL receptors, oxidative stress and apoptosis. Mol Cell Biochem.

[41] Greenshields AL (2015). Piperine inhibits the growth and motility of triple-negative breast cancer cells. Cancer Lett.

[42] Bashmail HA (2020). Thymoquinone enhances paclitaxel anti-breast cancer activity via inhibiting tumor-associated stem cells despite apparent mathematical antagonism. Molecules.

[43] Fatfat Z, Fatfat M, Gali-Muhtasib H (2021). Therapeutic potential of thymoquinone in combination therapy against cancer and cancer stem cells. World J Clin Oncol.

[44] Alkhatib MH, Bawadud RS, Gashlan HM (2020). Incorporation of docetaxel and thymoquinone in borage nanoemulsion potentiates their antineoplastic activity in breast cancer cells. Sci Rep.

[45] Jehan S (2020). Thymoquinone selectively induces hepatocellular carcinoma cell apoptosis in synergism with clinical therapeutics and dependence of p53 status. Front Pharmacol.

[46] Fattah A (2021). The Synergistic Combination of Cisplatin and Piperine Induces Apoptosis in MCF-7 Cell Line. Iran J Public Health.

[47] Jeong S (2020). Piperine synergistically enhances the effect of temozolomide against temozolomide-resistant human glioma cell lines. Bioengineered.

[48] Feitelson, M.A., et al. *Sustained proliferation in cancer: Mechanisms and novel therapeutic targets*. in *Seminars in cancer biology*. 2015. Elsevier. 10.1016/j.semcancer.2015.02.006 PMC4898971 25892662

[49] Fernando J (2012). Sorafenib sensitizes hepatocellular carcinoma cells to physiological apoptotic stimuli. J Cell Physiol.

[50] Dattachoudhury S (2020). Sorafenib inhibits proliferation, migration and invasion of breast cancer cells. Oncology.

[51] Youssef MM (2016). Novel combination of sorafenib and biochanin-A synergistically enhances the anti-proliferative and pro-apoptotic effects on hepatocellular carcinoma cells. Sci Rep.

[52] Sutton KM, Greenshields AL, Hoskin DW (2014). Thymoquinone, a bioactive component of black caraway seeds, causes G1 phase cell cycle arrest and apoptosis in triple-negative breast cancer cells with mutant p53. Nutr Cancer.

[53] Hassan S (2010). In vitro challenge using thymoquinone on hepatocellular carcinoma (HepG2) cell line. Iran J Pharm Res.

[54] Gali-Muhtasib HU (2004). Molecular pathway for thymoquinone-induced cell-cycle arrest and apoptosis in neoplastic keratinocytes. Anticancer Drugs.

[55] Rather RA, Bhagat M (2018). Cancer chemoprevention and piperine: molecular mechanisms and therapeutic opportunities. Front Cell  Dev Biol.

[56] Tutusaus A (2018). Antiapoptotic BCL-2 proteins determine sorafenib/regorafenib resistance and BH3-mimetic efficacy in hepatocellular carcinoma. Oncotarget.

[57] Jafri A (2019). Induction of apoptosis by piperine in human cervical adenocarcinoma via ROS mediated mitochondrial pathway and caspase-3 activation. EXCLI J.

[58] Hu X, Xuan Y (2008). Bypassing cancer drug resistance by activating multiple death pathways–a proposal from the study of circumventing cancer drug resistance by induction of necroptosis. Cancer Lett.

[59] Niederwieser C (2015). Prognostic and biologic significance of DNMT3B expression in older patients with cytogenetically normal primary acute myeloid leukemia. Leukemia.

[60] Zhou L (2018). Prognosis analysis of histone deacetylases mRNA expression in ovarian cancer patients. J Cancer.

[61] Cheray M (2015). DNMT inhibitors in cancer, current treatments and future promising approach: inhibition of specific DNMT-including complexes. Epigenetic Diagn  Ther.

[62] Suraweera A, O’Byrne KJ, Richard DJ (2018). Combination therapy with histone deacetylase inhibitors (HDACi) for the treatment of cancer: achieving the full therapeutic potential of HDACi. Front Oncol.

[63] Liang B-Y (2015). Synergistic suppressive effect of PARP-1 inhibitor PJ34 and HDAC inhibitor SAHA on proliferation of liver cancer cells. J Huazhong Univ Sci Technolog Med Sci.

[64] Venturelli S (2007). Epigenetic combination therapy as a tumor‐selective treatment approach for hepatocellular carcinoma*.*. Cancer.

[65] Abdullah O (2021). Thymoquinone Is a Multitarget Single Epidrug That Inhibits the UHRF1 Protein Complex. Genes.

[66] Bayat S (2018). HDACis (class I), cancer stem cell, and phytochemicals: Cancer therapy and prevention implications. Biomed Pharmacother.

[67] Garmpis N (2021). Histone Deacetylase Inhibitors in the Treatment of Hepatocellular Carcinoma: Current Evidence and Future Opportunities. J  Pers Med.

[68] Kwon JJ (2019). A systematic review of miR-29 in cancer. Mol Ther Oncolytics.

[69] Amodio N (2016). Therapeutic targeting of miR-29b/HDAC4 epigenetic loop in multiple myeloma. Mol Cancer Ther.

[70] Nagai M (2003). Expression of DNA (5-cytosin)-methyltransferases (DNMTs) in hepatocellular carcinomas. Hepatol Res.

[71] Oh B-K (2007). DNA methyltransferase expression and DNA methylation in human hepatocellular carcinoma and their clinicopathological correlation. Int J Mol Med.

[72] Robertson KD (1999). The human DNA methyltransferases (DNMTs) 1, 3a and 3b: coordinate mRNA expression in normal tissues and overexpression in tumors. Nucleic Acids Res.

[73] Liu Z (2009). Modulation of DNA methylation by a sesquiterpene lactone parthenolide. J Pharmacol Exp Ther.

[74] Lai S-C (2019). DNMT3b/OCT4 expression confers sorafenib resistance and poor prognosis of hepatocellular carcinoma through IL-6/STAT3 regulation. J Exp Clin Cancer Res.

[75] Audia JE, Campbell RM (2016). Histone modifications and cancer. Cold Spring Harb Perspect Biol.

[76] Parbin S (2014). Histone deacetylases: a saga of perturbed acetylation homeostasis in cancer. J Histochem Cytochem.

[77] Evans LW, Ferguson BS (2018). Food bioactive HDAC inhibitors in the epigenetic regulation of heart failure. Nutrients.

[78] Rajendran P (2011). Dietary phytochemicals, HDAC inhibition, and DNA damage/repair defects in cancer cells. Clin Epigenetics.

[79] Eckschlager T (2017). Histone deacetylase inhibitors as anticancer drugs. Int J Mol Sci.

[80] Maolanon AR, Madsen AS, Olsen CA (2016). Innovative strategies for selective inhibition of histone deacetylases. Cell Chem Biol.

[81] Frew AJ, Johnstone RW, Bolden JE (2009). Enhancing the apoptotic and therapeutic effects of HDAC inhibitors. Cancer Lett.

