# Supplementary material for: Thymoquinone, piperine, and sorafenib combinations attenuate liver and breast cancers progression: epigenetic and molecular docking approaches
Source: BMC Complement Med Ther. 2023 Mar 4;23:69. doi: 10.1186/s12906-023-03872-6 (PMC9985300; doi:10.1186/s12906-023-03872-6)
Supplement: Supplementary file 2 — Additional file 2. Cancer clinical trials of sorafenib. [file 12906_2023_3872_MOESM2_ESM.pdf]

Supplementary File 2: Cancer clinical trials of Sorafenib

ClinicalTrials.gov Search Results 07/28/2022

	Title	Status	Study Results	Conditions	Interventions	Locations
1	Dose Escalation Study of OMP-54F28 in Combination With Sorafenib in Patients With Hepatocellular Cancer	Completed	No Results Available	•Hepatocellular Cancer<br>•Liver Cancer	•Drug: OMP-54F28 with Sorafenib	•USC/Norris Comprehensive Cancer Center, Los Angeles, California, United States<br>•University of Colorado Cancer Center, Aurora, Colorado, United States<br>•Indiana University Simon Cancer Center, Indianapolis, Indiana, United States<br>•Massachussetts General Hospital, Boston, Massachusetts, United States<br>•Mount Sinai Medical Center, New York, New York, United States<br>•Fox Chase Cancer Center, Philadelphia, Pennsylvania, United States
2	A Phase II Trial of OSI-906 and Sorafenib in Advanced Hepatocellular Cancer	Terminated	Has Results	•Liver Cancer	•Drug: Sorafenib and OSI-906	•Emory University Winship Cancer Institute, Atlanta, Georgia, United States<br>•University of Michigan, Ann Arbor, Michigan, United States<br>•Ohio State University, Columbus, Ohio, United States
3	Sorafenib Plus Capecitabine (SorCape) in Previously Treated Metastatic Colorectal Cancer	Completed	Has Results	•Colorectal Cancer Metastatic	•Drug: Sorafenib Plus Capecitabine (SorCape)	•UF Health Cancer Center, Gainesville, Florida, United States
4	Preoperative Trial of Sorafenib in Combination With Cisplatin Followed by Paclitaxel for Early Stage Breast Cancer	Terminated	Has Results	•Breast Cancer	•Drug: Sorafenib<br>•Drug: Cisplatin<br>•Drug: Paclitaxel	•Grady Memorial Hospital, Atlanta, Georgia, United States<br>•Emory University Hospital Midtown, Atlanta, Georgia, United States<br>•Emory University Winship Cancer Institute, Atlanta, Georgia, United States
5	Safety and Efficacy of Sorafenib in Patients With Advanced Thyroid Cancer: a Phase II Clinical Study	Completed	No Results Available	•Thyroid Cancer	•Drug: Sorafenib	•Instituto Nacional de Cancerología, Bogotá, Cundinamarca, Colombia
6	BAY43-9006 - Phase II in Advanced Breast Cancer	Completed	Has Results	•Breast Neoplasms<br>•Breast Cancer	•Drug: Sorafenib (Nexavar, BAY43-9006)	•Stuttgart, Baden-Württemberg, Germany<br>•München, Bayern, Germany<br>•Frankfurt, Hessen, Germany<br>•Bologna, Italy<br>•Milano, Italy<br>•Milano, Italy<br>•Parma, Italy
7	Sorafenib and Vinorelbine in Treating Women With Stage IV Breast Cancer	Completed	Has Results	•Breast Cancer	•Drug: sorafenib tosylate<br>•Drug: vinorelbine ditartrate	•City of Hope Medical Center, Duarte, California, United States<br>•South Pasadena Cancer Center, Pasadena, California, United States
8	Phase II Trial of Vemurafenib and Sorafenib in Pancreatic Cancer	Recruiting	No Results Available	•Pancreas Cancer	•Drug: Vemurafenib<br>•Drug: Sorafenib	•HonorHealth Research Institute, Scottsdale, Arizona, United States
9	Phase I/II Trial of Letrozole and Sorafenib in Post-Menopausal Hormone-Receptor + Breast Cancer	Terminated	No Results Available	•Metastatic Breast Cancer	•Drug: Sorafenib<br>•Drug: Letrozole	•Georgetown University Hospital, Washington, District of Columbia, United States<br>•Emory University, Atlanta, Georgia, United States<br>•Rutgers Cancer Institute of New Jersey, New Brunswick, New Jersey, United States<br>•Cooper Hospital/University Medical Center, Voorhees, New Jersey, United States

	Title	Status	Study Results	Conditions	Interventions	Locations
10	Sorafenib and Paclitaxel in Treating Patients With Metastatic Breast Cancer	Terminated	Has Results	•Breast Cancer	•Drug: paclitaxel<br>•Drug: sorafenib tosylate	•University of Texas Southwestern Medical Center, Dallas, Texas, United States
11	Trial of Sorafenib in Hepatocellular Cancer (HCC) Transplant Patients	Completed	No Results Available	•Hepatocellular Cancer	•Drug: Sorafenib	•University of Southern California, Los Angeles, California, United States<br>•University of California, Los Angeles, Los Angeles, California, United States<br>•Columbia University Medical Center, New York, New York, United States
12	Study of Everolimus and Sorafenib in Patients With Advanced Thyroid Cancer Who Progressed on Sorafenib Alone	Active, not recruiting	No Results Available	•Differentiated Thyroid Cancer	•Drug: Everolimus<br>•Drug: Sorafenib	•Hospital of the University of Pennsylvania, Philadelphia, Pennsylvania, United States
13	Safety Study of Sorafenib With Androgen Deprivation and Radiotherapy to Treat Prostate Cancer	Terminated	Has Results	•Prostate Cancer	•Drug: Leuprolide acetate, Bicalutamide, Sorafenib	•St.Luke's-Roosevelt Hospital Center, New York, New York, United States
14	Study Adding Multikinase Inhibitor Sorafenib to Existing Endocrine Therapy in Patients With Advanced Breast Cancer	Terminated	Has Results	•Breast Cancer	•Drug: sorafenib	•University of Kentucky, Lexington, Kentucky, United States
15	Sorafenib Induced Autophagy Using Hydroxychloroquine in Hepatocellular Cancer	Recruiting	No Results Available	•Hepatocellular Cancer	•Drug: Sorafenib (SOR)<br>•Drug: Hydroxychloroquine (HCQ)	•University of Texas Health Cancer Center, San Antonio, Texas, United States
16	A Phase I Trial Evaluating mFOLFOX6 and Avastin With Nexavar as First-Line Treatment for Metastatic Colorectal Cancer	Terminated	Has Results	•Metastatic Colorectal Cancer	•Drug: sorafenib<br>•Drug: bevacizumab<br>•Drug: mFOLFOX6 regimen	•Central Georgia Cancer Care, Macon, Georgia, United States<br>•Hematology Oncology Centers of the Northern Rockies, Billings, Montana, United States<br>•The West Clinic, Memphis, Tennessee, United States
17	Paclitaxel and Carboplatin With Or Without Sorafenib In The First-Line Treatment Of Patients With Ovarian Cancer	Completed	Has Results	•Ovarian Cancer	•Drug: Sorafenib<br>•Drug: Paclitaxel<br>•Drug: Carboplatin	•Northeast Arkansas Clinic, Jonesboro, Arkansas, United States<br>•Florida Cancer Specialists, Fort Myers, Florida, United States<br>•Holy Cross Hospital, Ft. Lauderdale, Florida, United States<br>•Gulfcoast Oncology Associates, St. Petersburg, Florida, United States<br>•Medical College of Georgia Cancer Specialists, Augusta, Georgia, United States<br>•Providence Medical Group, Terre Haute, Indiana, United States<br>•Center for Cancer and Blood Disorders, Bethesda, Maryland, United States<br>•National Capital Clinical Research Consortium, Bethesda, Maryland, United States<br>•St. Joseph Mercy Hospital, Ann Arbor, Michigan, United States<br>•Grand Rapids Clinical Oncology Program, Grand Rapids, Michigan, United States<br>•and 7 more

	Title	Status	Study Results	Conditions	Interventions	Locations
18	Phase III Trial Comparing Capecitabine in Combination With Sorafenib or Placebo in the Treatment of Locally Advanced or Metastatic HER2-Negative Breast Cancer	Completed	Has Results	•Breast Cancer	•Drug: Sorafenib (Nexavar, BAY43-9006)<br>•Drug: Placebo<br>•Drug: Capecitabine	•Greenbrae, California, United States<br>•Sylmar, California, United States<br>•West Palm Beach, Florida, United States<br>•Joliet, Illinois, United States<br>•Evansville, Indiana, United States<br>•Louisville, Kentucky, United States<br>•Boston, Massachusetts, United States<br>•Boston, Massachusetts, United States<br>•Jackson, Mississippi, United States<br>•Springfield, Missouri, United States<br>•and 143 more
19	Sorafenib Tosylate Before and After Hepatic Arterial Chemoembolization With Doxorubicin Hydrochloride and Mitomycin C in Treating Patients With Localized Liver Cancer That Cannot Be Removed by Surgery	Completed	No Results Available	•Liver Cancer	•Drug: sorafenib tosylate, HACE : Doxorubicin Hydrochloride and Mitomycin C<br>•Other: laboratory biomarker analysis	•Rutgers University Hospital, Newark, New Jersey, United States
20	Sorafenib and Docetaxel in Patients With Prostate Cancer That Did Not Respond to Previous Hormone Therapy	Completed	Has Results	•Prostate Cancer	•Drug: docetaxel<br>•Drug: sorafenib tosylate	•Abramson Cancer Center of the University of Pennsylvania, Philadelphia, Pennsylvania, United States
21	Study Combining Imatinib Mesylate (Gleevec) With Sorafenib in Patients With Androgen-independent Prostate Cancer (AIPC)	Completed	Has Results	•Prostate Cancer	•Drug: Gleevec + Sorafenib	•Oncology Specialists, S.C, Niles, Illinois, United States<br>•Oncology Specialists, S.C, Park Ridge, Illinois, United States
22	Sorafenib and Fulvestrant in Treating Patients With Locally Advanced or Metastatic Breast Cancer That Did Not Respond to Aromatase Inhibitor Therapy	Terminated	Has Results	•Breast Cancer	•Drug: fulvestrant<br>•Drug: sorafenib tosylate	•OHSU Knight Cancer Institute, Portland, Oregon, United States
23	A Phase II Study of BAY 43-9006 (Sorafenib) in Metastatic, Androgen-Independent Prostate Cancer	Completed	Has Results	•Prostate Cancer	•Drug: BAY 43-9006	•National Cancer Institute (NCI), Bethesda, Maryland, United States
24	External-Beam Radiation Therapy, Capecitabine, and Sorafenib in Treating Patients With Locally Advanced Rectal Cancer	Completed	No Results Available	•Colorectal Cancer	•Drug: capecitabine<br>•Drug: sorafenib tosylate<br>•Radiation: radiation therapy	•Szent Laszlo Korhaz, Budapest, Hungary<br>•Saint Claraspital AG, Basel, Switzerland<br>•Universitaetsspital-Basel, Basel, Switzerland<br>•Istituto Oncologico della Svizzera Italiana - Ospedale Regionale Bellinzona e Valli, Bellinzona, Switzerland<br>•Inselspital, Bern, Bern, Switzerland<br>•Spitalzentrum Biel, Biel, Switzerland<br>•Kantonsspital Bruderholz, Bruderholz, Switzerland<br>•Kantonsspital Graubuenden, Chur, Switzerland<br>•Hopital Cantonal Universitaire de Geneva HUG, Geneva, Switzerland<br>•Kantonsspital Luzern, Luzern, Switzerland<br>•and 8 more

	Title	Status	Study Results	Conditions	Interventions	Locations
25	Phase I/II Trial of Sorafenib Plus Ixabepilone in HER2-Negative Metastatic Breast Cancer	Completed	Has Results	•Metastatic Breast Cancer	•Drug: Sorafenib<br>•Drug: Ixabepilone	•Florida Cancer Specialists, Ft. Myers, Florida, United States<br>•Providence Medical Group, Terre Haute, Indiana, United States<br>•RHHP/ Hope Cancer Center, Terre Haute, Indiana, United States<br>•Baptist Hospital East, Louisville, Kentucky, United States<br>•Hematology Oncology Clinic, LLP, Baton Rouge, Louisiana, United States<br>•Mercy Hospital, Portland, Maine, United States<br>•Center for Cancer and Blood Disorders, Bethesda, Maryland, United States<br>•National Capital Clinical Research Consortium, Bethesda, Maryland, United States<br>•St. Louis Cancer Care, Chesterfield, Missouri, United States<br>•Portsmouth Regional Hospital, Portsmouth, New Hampshire, United States<br>•and 5 more
26	Sorafenib in Treating Patients With Refractory Non-Small Cell Lung Cancer	Completed	Has Results	•Lung Cancer	•Drug: Sorafenib<br>•Drug: Placebo	•Stanford Comprehensive Cancer Center - Stanford, Stanford, California, United States<br>•Aurora Presbyterian Hospital, Aurora, Colorado, United States<br>•Boulder Community Hospital, Boulder, Colorado, United States<br>•Penrose Cancer Center at Penrose Hospital, Colorado Springs, Colorado, United States<br>•Porter Adventist Hospital, Denver, Colorado, United States<br>•Presbyterian - St. Luke's Medical Center, Denver, Colorado, United States<br>•St. Joseph Hospital, Denver, Colorado, United States<br>•Rose Medical Center, Denver, Colorado, United States<br>•CCOP - Colorado Cancer Research Program, Denver, Colorado, United States<br>•Swedish Medical Center, Englewood, Colorado, United States<br>•and 131 more
27	Sorafenib in Treating Non-Smokers or Former Light Smokers With Relapsed or Refractory Stage IIIB or Stage IV Non-Small Cell Lung Cancer	Terminated	Has Results	•Lung Cancer	•Drug: sorafenib	•Ohio State University, Columbus, Ohio, United States

	Title	Status	Study Results	Conditions	Interventions	Locations
28	Sorafenib in Treating Patients With Kidney Cancer That Has Spread to the Brain	Completed	No Results Available	•Kidney Cancer<br>•Metastatic Cancer	•Drug: sorafenib tosylate	•University of Chicago Cancer Research Center, Chicago, Illinois, United States<br>•Decatur Memorial Hospital Cancer Care Institute, Decatur, Illinois, United States<br>•Evanston Northwestern Health Care - Evanston Hospital, Evanston, Illinois, United States<br>•Ingalls Cancer Care Center at Ingalls Memorial Hospital, Harvey, Illinois, United States<br>•Cardinal Bernardin Cancer Center at Loyola University Medical Center, Maywood, Illinois, United States<br>•Oncology Hematology Associates of Central Illinois, PC - Peoria, Peoria, Illinois, United States<br>•Central Illinois Hematology Oncology Center, Springfield, Illinois, United States<br>•Fort Wayne Medical Oncology and Hematology, Fort Wayne, Indiana, United States<br>•CCOP - Northern Indiana CR Consortium, South Bend, Indiana, United States<br>•Oncology Care Associates, PLLC, Saint Joseph, Michigan, United States<br>•David C. Pratt Cancer Center at St. John's Mercy, Saint Louis, Missouri, United States<br>•Medical College of Wisconsin Cancer Center, Milwaukee, Wisconsin, United States
29	Sorafenib and Radiotherapy in the Treatment of Invasive Bladder Cancer With Conservative Intent	Completed	No Results Available	•Bladder Cancer	•Drug: Sorafenib<br>•Procedure: Radiotherapy	•Hospital Clínic i Provincial de Barcelona, Barcelona, Spain<br>•Hospital de la Santa Creu i Sant Pau, Barcelona, Spain<br>•Institut Català d' Oncología, Barcelona, Spain
30	Sorafenib Plus Capecitabine Efficacy Assessment in Patients With Advanced Pre-treated Colorectal Cancer	Completed	No Results Available	•Colorectal Cancer	•Drug: chemotherapy	•Cliniques Universitaires Saint Luc, Woluwe Saint Lambert, Bruxelles, Belgium<br>•Universiteit Ziekenhuis Antwerpen, Antwerpen, Belgium<br>•Institut Jules Bordet, Brussels, Belgium<br>•Hopital Erasme, Brussels, Belgium<br>•Entité Jolimontoise, La Louviere, Belgium<br>•Hopitaux St Joseph, Liege, Belgium<br>•CHU Ambroise Paré, Mons, Belgium
31	Sorafenib and Thoracic Radiation for Patients With Non-Small Cell Lung Cancer	Terminated	No Results Available	•Lung Cancer	•Drug: Sorafenib<br>•Procedure: Radiation Therapy	•UT MD . Anderson Cancer Center, Houston, Texas, United States
32	Phase IB/II Trial Combining Vinorelbine With Sorafenib as First-Line Treatment in Patients With Metastatic Breast Cancer	Unknown status	No Results Available	•Metastatic Breast Cancer	•Drug: Sorafenib and Vinorelbine	•Jewish General Hospital, Montreal, Quebec, Canada
33	Sorafenib Concurrent With Yttrium-90 Transarterial Radioembolization in Patients With Advanced Hepatocellular Cancer	Withdrawn	No Results Available	•Hepatocellular Cancer	•Drug: Sorafenib<br>•Radiation: yttrium-90 radioembolization	•University of Hawaii, Honolulu, Hawaii, United States
34	Phase I Trial of Sorafenib + FOLFIRI In Metastatic Colorectal Cancer	Completed	No Results Available	•Metastatic Colorectal Cancer	•Drug: sorafenib + FOLFIRI	•The Ottawa Hospital Cancer Centre, Ottawa, Ontario, Canada

	Title	Status	Study Results	Conditions	Interventions	Locations
35	Evaluating the Combination of Everolimus and Sorafenib in the Treatment of Thyroid Cancer	Active, not recruiting	No Results Available	• Thyroid Cancer	• Drug: sorafenib with everolimus	• Memoral Sloan Kettering Cancer Center, Basking Ridge, New Jersey, United States<br>• Memorial Sloan Kettering Cancer Center @ Suffolk, Commack, New York, United States<br>• Memorial Sloan Kettering Westchester, Harrison, New York, United States<br>• Memorial Sloan Kettering Cancer Center, New York, New York, United States
36	Trametinib in Combination With Sorafenib in Patients With Advanced Hepatocellular Cancer	Completed	No Results Available	• Hepatocellular Cancer<br>• Liver Cancer	• Drug: Trametinib<br>• Drug: Sorafenib	• H. Lee Moffitt Cancer Center and Research Institute, Tampa, Florida, United States
37	Breast Cancer Study: Paclitaxel Versus Paclitaxel Plus Sorafenib in Second- or Third-line Treatment	Completed	No Results Available	• Metastatic Breast Cancer	• Drug: Paclitaxel and Sorafenib<br>• Drug: Paclitaxel	• Germany Multiple Sites All Over Germany Recruiting Multiple Sites, Germany Contact: iOMEDICO AG, Freiburg, Germany
38	Sorafenib and Erlotinib in Treating Patients With Pancreatic Cancer That Cannot Be Removed by Surgery	Completed	Has Results	• Pancreatic Cancer	• Drug: Sorafenib<br>• Drug: Erlotinib	• Purchase Cancer Group - Paducah, Paducah, Kentucky, United States<br>• Erlanger Cancer Center at Erlanger Hospital - Baroness, Chattanooga, Tennessee, United States<br>• Baptist Regional Cancer Center at Baptist Riverside, Knoxville, Tennessee, United States<br>• Vanderbilt-Ingram Cancer Center - Cool Springs, Nashville, Tennessee, United States<br>• Vanderbilt-Ingram Cancer Center at Franklin, Nashville, Tennessee, United States<br>• Vanderbilt-Ingram Cancer Center, Nashville, Tennessee, United States
39	Mitoxantrone, Prednisone Plus Sorafenib in Taxane-Refractory Metastatic Hormone Refractory Prostate Cancer (HRPC)	Terminated	Has Results	• Metastatic Prostate Cancer	• Drug: Mitoxantrone<br>• Drug: Prednisone<br>• Drug: Sorafenib	• Wilshire Oncology Medical Group, Inc., La Verne, California, United States<br>• Peachtree Hematology Oncology Consultants, Atlanta, Georgia, United States<br>• Central Georgia Cancer Care, Macon, Georgia, United States<br>• Northwest Georgia Oncology Centers, Marietta, Georgia, United States<br>• Hematology Oncology Centers of the Northern Rockies, PC, Billings, Montana, United States<br>• Mid-Ohio Oncology/Hematology, Inc., Columbus, Ohio, United States<br>• Lancaster Cancer Center, Lancaster, Pennsylvania, United States<br>• Pennsylvania Oncology Hematmology Associates, Philadelphia, Pennsylvania, United States<br>• The West Clinic, Memphis, Tennessee, United States<br>• Cancer Specialists of Tidewater, Chesapeake, Virginia, United States
40	Sorafenib to Overcome Resistance to Systemic Chemotherapy in Androgen-independent Prostate Cancer	Completed	Has Results	• Prostate Cancer	• Drug: Sorafenib	• Onocology Specialists, S.C, Niles, Illinois, United States<br>• Oncology Specialists, S.C, Park Ridge, Illinois, United States

	Title	Status	Study Results	Conditions	Interventions	Locations
41	Sorafenib in Treating Patients With Metastatic or Recurrent Prostate Cancer	Completed	No Results Available	•Prostate Cancer	•Drug: sorafenib tosylate	•Tom Baker Cancer Centre - Calgary, Calgary, Alberta, Canada<br>•British Columbia Cancer Agency - Centre for the Southern Interior, Kelowna, British Columbia, Canada<br>•British Columbia Cancer Agency - Vancouver Cancer Centre, Vancouver, British Columbia, Canada<br>•CancerCare Manitoba, Winnipeg, Manitoba, Canada<br>•Margaret and Charles Juravinski Cancer Centre, Hamilton, Ontario, Canada<br>•London Regional Cancer Program at London Health Sciences Centre, London, Ontario, Canada<br>•Princess Margaret Hospital, Toronto, Ontario, Canada
42	Nexavar for Neoadjuvant Treatment of Anaplastic Thyroid Cancer	Unknown status	No Results Available	•Thyroid Cancer, Anaplastic	•Drug: Nexavar<br>•Procedure: operation<br>•Radiation: External radiation therapy	•Fujian Medical University Union Hospital, FuZhou, Fujian, China
43	Sorafenib, Cisplatin, and Etoposide in Treating Patients With Extensive-Stage Small Cell Lung Cancer	Terminated	Has Results	•Lung Cancer	•Drug: cisplatin<br>•Drug: etoposide<br>•Drug: sorafenib tosylate	•Columbia Presbyterian, New York, New York, United States<br>•Lake/University Ireland Cancer Center, Cleveland, Ohio, United States<br>•Case Medical Center, University Hospitals Seidman Cancer Center, Case Comprehensive Cancer Center, Cleveland, Ohio, United States<br>•MetroHealth Medical Center, Cleveland, Ohio, United States<br>•CCF-Fairview Hospital, Cleveland, Ohio, United States<br>•UHHS Chagrin Highlands Medical Center, Cleveland, Ohio, United States<br>•Southwest General Health Center, Cleveland, Ohio, United States<br>•UHHS Westlake Medical Center, Cleveland, Ohio, United States<br>•Cleveland Clinic Taussig Cancer Institute, Case Comprehensive Cancer Center, Cleveland, Ohio, United States<br>•UH-Monarch, Mayfield Heights, Ohio, United States<br>•UH-Firelands, Sandusky, Ohio, United States
44	Study of Sorafenib and Docetaxel in Metastatic Prostate Cancer	Completed	No Results Available	•Prostate Cancer	•Drug: Sorafenib (Nexavar)<br>•Drug: Docetaxel	•Dipartimento di Oncologia, dei Trapianti e delle Nuove Tecnologie in Medicina ... Indirizzo: via Roma, 55 - 56100 Pisa Tel. 050-2218690 - Fax. 050-2218685, Pisa, Italy
45	Combination of Temsirolimus and Sorafenib in the Treatment of Radioactive Iodine Refractory Thyroid Cancer	Completed	Has Results	•Thyroid Cancer	•Drug: Temsirolimus and Sorafenib	•Memorial Sloan-Kettering at Basking Ridge, Basking Ridge, New Jersey, United States<br>•Memorial Sloan-Kettering Cancer Center @ Suffolk, Commack, New York, United States<br>•Memorial Sloan Kettering Cancer Center, New York, New York, United States<br>•Memorial Sloan-Kettering at Mercy Medical Center, Rockville Centre, New York, United States<br>•Memorial Sloan-Kettering Cancer Center at Phelps Memorial Hospital Center, Sleepy Hollow, New York, United States
46	Sorafenib Treatment in Non-Small Cell Lung Cancer After Failure of Epidermal Growth Factor Receptor-tyrosine Kinase Inhibitor	Completed	No Results Available	•Non-Small Cell Lung Cancer	•Drug: sorafenib (Nexavar)	•WuYiLong, Guangzhou, Guangdong, China

	Title	Status	Study Results	Conditions	Interventions	Locations
47	A Phase I/II Study of Cisplatin and Radiation in Combination With Sorafenib in Cervical Cancer	Completed	No Results Available	•Cancer of the Cervix	•Drug: Sorafenib<br>•Drug: Cisplatin<br>•Procedure: Radiation	•Princess Margaret Hospital, Toronto, Ontario, Canada
48	Sorafenib in Combination With Irinotecan in Metastatic Colorectal Cancer Patients With KRAS Mutated Tumors	Completed	No Results Available	•Metastatic Colorectal Cancer Patients With KRAS Mutated Tumors	•Drug: Sorafenib and irinotecan combination<br>•Drug: Sorafenib monotherapy<br>•Drug: Irinotecan monotherapy	•Hôpital AVICENNE, Bobigny, France<br>•Centre François Baclesse, Caen, France<br>•Centre Oscar Lambret, Lille, France<br>•Centre Léon Bérard, Lyon, France<br>•Hôpital LA TIMONE, Marseille, France<br>•CRLC Val d'Aurelle-Paul Lamarque, Montpellier, France<br>•C.H.U. de REIMS, Reims, France<br>•CHU Charles Nicolle, Rouen, France<br>•Institut de Cancérologie de l'Ouest - René Gauducheau, St. Herblain, France
49	Letrozole Plus Oral Cyclophosphamide Plus/Minus Sorafenib as Primary Systemic Treatment in Breast Cancer Patients	Unknown status	No Results Available	•Breast Cancer	•Drug: letrozolo+cyclophosphamide<br>•Drug: letrozolo+sorafenib +cyclophosphamide	•Breast Unit Azienda Ospedaliera Istituti Ospitalieri, Cremona, Italy<br>•Azienda Ospedaliera S.Luigi Orbassano, Orbassano, Italy
50	Sorafenib as a Second Line Treatment in Patients With Advanced or Metastatic Gastric Cancer	Terminated	No Results Available	•Gastric Cancer	•Drug: sorafenib	•Fudan University Cancer Hospital, ShangHai, Shanghai, China
51	Sorafenib Therapy Prior to Radiofrequency Ablation for Intermediate Sized Hepatocellular Cancer	Completed	Has Results	•Hepatocellular Cancer	•Drug: Sorafenib<br>•Procedure: radiofrequency ablation	•Dana-Farber Cancer Institute, Boston, Massachusetts, United States<br>•Beth Israel Deaconess Medical Center, Boston, Massachusetts, United States
52	Phase I Study of Perifosine + Sorafenib for Patients With Advanced Cancers	Completed	No Results Available	•Renal Cancer<br>•Tumors	•Drug: Perifosine<br>•Drug: Sorafenib	
53	Capecitabine and Cisplatin (XP)+Sorafenib in Advanced Gastric Cancer (AGC): Sorafenib+XP	Completed	Has Results	•Advanced Gastric Cancer	•Drug: Capecitabine, Cisplatin, Sorafenib	•Asan Medical Center, Seoul, Korea, Republic of
54	Erlotinib and Sorafenib in Chemonaive Patients With Locally Advanced or Metastatic Non Small Cell Lung Cancer	Unknown status	No Results Available	•Non-Small Cell Lung Cancer	•Drug: sorafenib + erlotinib	•VU university medical center, Amsterdam, Netherlands<br>•Netherlands Cancer Institute, Amsterdam, Netherlands<br>•University Medical Center Groningen, Groningen, Netherlands<br>•University Hospital Maastricht, Maastricht, Netherlands

	Title	Status	Study Results	Conditions	Interventions	Locations
55	A Phase II Study of Bevacizumab + Sorafenib in Metastatic Breast Cancer	Terminated	Has Results	•Metastatic Breast Cancer	•Drug: Sorafenib<br>•Drug: Bevacizumab<br>•Other: Imaging	•Medical & Surgical Specialists, LLC, Galesburg, Illinois, United States<br>•Oncology Hematology Associates of SW Indiana, Evansville, Indiana, United States<br>•Fort Wayne Oncology & Hematology, Inc, Fort Wayne, Indiana, United States<br>•Indiana University Simon Cancer Center, Indianapolis, Indiana, United States<br>•Quality Cancer Center (MCGOP), Indianapolis, Indiana, United States<br>•Arnett Cancer Care, Lafayette, Indiana, United States<br>•Horizon Oncology Center, Lafayette, Indiana, United States<br>•Medical Consultants, P.C., Muncie, Indiana, United States<br>•Northern Indiana Cancer Research Consortium, South Bend, Indiana, United States<br>•Ireland Cancer Center - University Hospitals of Cleveland, Cleveland, Ohio, United States
56	Sorafenib in Hormone Naïve Biochemical Recurrence of Prostate Cancer	Withdrawn	No Results Available	•Prostate Cancer	•Drug: Sorafenib<br>•Drug: Placebo	•Fox Chase Cancer Center, Philadelphia, Pennsylvania, United States
57	Sorafenib and Anastrozole in Treating Postmenopausal Women With Metastatic Breast Cancer	Completed	Has Results	•Recurrent Breast Cancer<br>•Stage IV Breast Cancer	•Drug: sorafenib tosylate<br>•Drug: anastrozole	•Lombardi Comprehensive Cancer Center at Georgetown University, Washington, District of Columbia, United States
58	Sorafenib Tosylate in Treating Patients With Liver Cancer That Can Be Removed by Surgery	Unknown status	No Results Available	•Liver Cancer	•Drug: sorafenib tosylate<br>•Other: laboratory biomarker analysis<br>•Procedure: neoadjuvant therapy<br>•Procedure: therapeutic conventional surgery	•Hopital Beaujon, Clichy, France
59	A Randomized Phase II Trial of Surgery Plus Sorafenib vs. Sorafenib Alone for Hepatocellular Cancer (HCC) With Portal Vein Invasion	Recruiting	No Results Available	•Hepatocellular Cancer	•Procedure: hepatic resection<br>•Drug: Sorafenib	•Eastern Hepatobiliary Surgery Hospital, Shanghai, China
60	Sorafenib and Erlotinib or Sorafenib Alone in Advanced Non-Small Cell Lung Cancer Progressing on Erlotinib	Completed	Has Results	•Non-Small Cell Lung Cancer	•Drug: Sorafenib<br>•Drug: Erlotinib	•Florida Cancer Specialists, Fort Myers, Florida, United States<br>•Northeast Georgia Medical Center, Gainesville, Georgia, United States<br>•Wellstar Cancer Research, Marietta, Georgia, United States<br>•Providence Medical Group, Terre Haute, Indiana, United States<br>•Norton Cancer Institute, Louisville, Kentucky, United States<br>•Hematology Oncology Clinic, LLP, Baton Rouge, Louisiana, United States<br>•Center for Cancer and Blood Disorders, Bethesda, Maryland, United States<br>•National Capital Clinical Research Consortium, Bethesda, Maryland, United States<br>•Jackson Oncology Associates, Jackson, Mississippi, United States<br>•St. Louis Cancer Care, Chesterfield, Missouri, United States<br>•and 6 more

	Title	Status	Study Results	Conditions	Interventions	Locations
61	A Study of Combination of Gemcitabine, Oxaliplatin (GEMOX)-Sorafenib in Patients With Advanced Biliary Tract Cancer	Terminated	Has Results	•Cholangiocarcinoma<br>•Biliary Tract Cancer<br>•Gallbladder Cancer	•Drug: Gemcitabine<br>•Drug: Oxaliplatin<br>•Drug: Sorafenib	•University of Miami, Miami, Florida, United States
62	BAY 43-9006 Plus Cetuximab to Treat Colorectal Cancer	Completed	Has Results	•Metastatic Colorectal Cancer	•Drug: Cetuximab<br>•Drug: BAY 43-9006	•National Institutes of Health Clinical Center, 9000 Rockville Pike, Bethesda, Maryland, United States
63	Sorafenib Gastric Cancer Asian Phase I Study	Completed	No Results Available	•Gastric Cancer	•Drug: Sorafenib (Nexavar, BAY43-9006)	•Kashiwa, Chiba, Japan<br>•Kobe, Hyogo, Japan<br>•Chuo-ku, Tokyo, Japan
64	Sorafenib and Letrozole, Anastrozole, or Exemestane in Treating Postmenopausal Women With Estrogen Receptor-Positive and/or Progesterone Receptor-Positive Metastatic Breast Cancer	Terminated	Has Results	•Breast Cancer	•Drug: anastrozole<br>•Drug: exemestane<br>•Drug: letrozole<br>•Drug: sorafenib tosylate<br>•Other: placebo	•Mayo Clinic in Arizona, Scottsdale, Arizona, United States<br>•Mayo Clinic in Florida, Jacksonville, Florida, United States<br>•Mayo Clinic, Rochester, Minnesota, United States
65	Pilot Study of Sorafenib and Bi-weekly Capecitabine in Patients With Advanced Breast and Gastrointestinal Tumors	Completed	No Results Available	•Metastatic Breast Cancer<br>•Gastrointestinal Tumors	•Drug: Sorafenib<br>•Drug: Capecitabine	•Yale Cancer Center, New Haven, Connecticut, United States
66	Sorafenib and Vorinostat in Treating Patients With Advanced Liver Cancer	Completed	No Results Available	•Liver Cancer	•Drug: sorafenib tosylate<br>•Drug: vorinostat	•Hunter Holmes McGuire VA Medical Center, Richmond, Virginia, United States<br>•Virginia Commonwealth University/Massey Cancer Center, Richmond, Virginia, United States
67	Efficacy and Safety Study of Sorafenib With Topotecan in Patients With Platinum-resistant Recurrent Ovarian Cancer	Completed	No Results Available	•Ovarian Cancer	•Drug: Sorafenib	•Charité Campus Virchow-Klinikum, Berlin, Germany
68	Sorafenib With Irinotecan in Metastatic Colorectal Cancer (mCRC) and K-RAS Mutation	Completed	No Results Available	•Metastatic Colorectal Cancer	•Drug: Nexavar (Sorafenib) and irinotecan (Campto)	•Centre Oscar Lambret, Lille, France<br>•Hopital Saint Eloi, Montpellier, France<br>•Centre Rene Gauducheau, Nantes, France<br>•Centre Antoine Lacassagne, Nice, France<br>•CHU Robert Debre, Reims, France
69	Sorafenib and Bevacizumab to Treat Ovarian, Fallopian and Peritoneal Cancer	Completed	Has Results	•Ovarian Neoplasm<br>•Fallopian Tube Cancer<br>•Primary Peritoneal Cancer	•Drug: Bevacizumab<br>•Drug: BAY 43-9006	•National Institutes of Health Clinical Center, 9000 Rockville Pike, Bethesda, Maryland, United States
70	Pharmacokinetic Study of BAY43-9006 and Taxotere to Treat Patient With Prostatic Cancer	Completed	No Results Available	•Primary Disease<br>•Prostate Cancer	•Drug: sorafenib (200 or 400mg bid) and taxotere iv	•St Pierre, Ottignies, Brabant Wallon, Belgium<br>•Cliniques Universitaires St Luc, Brussels, Brussels Capital, Belgium<br>•Notre Dame et Reine Fabiola, Charleroi, Hainaut, Belgium<br>•Clinique Universiataire de Mont Godinne, Yvoir, Namur, Belgium<br>•Sainte Elisabeth, Namur, Belgium<br>•Hôpital Européen Georges Pompidou, Paris, France

	Title	Status	Study Results	Conditions	Interventions	Locations
71	Adjuvant Doxorubicin/Cyclophosphamide and Paclitaxel Plus Sorafenib Breast Cancer	Completed	Has Results	•Breast Cancer	•Drug: Doxorubicin<br>•Drug: Cyclophosphamide<br>•Drug: Paclitaxel<br>•Drug: Sorafenib	•Integrated Community Oncology Network, Jacksonville, Florida, United States<br>•Florida Hospital Cancer Institute, Orlando, Florida, United States<br>•Consultants in Blood Disorders and Cancer, Louisville, Kentucky, United States<br>•Grand Rapids Clinical Oncology Program, Grand Rapids, Michigan, United States<br>•Methodist Cancer Center, Omaha, Nebraska, United States<br>•Chattanooga Oncology Hematology Associates, Chattanooga, Tennessee, United States<br>•Tennessee Oncology, PLLC, Nashville, Tennessee, United States<br>•Peninsula Cancer Institute, Newport News, Virginia, United States
72	Sorafenib Tosylate With or Without Everolimus in Treating Patients With Advanced, Radioactive Iodine Refractory Hurthle Cell Thyroid Cancer	Active, not recruiting	Has Results	•Refractory Hurthle Cell Thyroid Cancer	•Drug: sorafenib<br>•Drug: everolimus	•Mayo Clinic in Florida, Jacksonville, Florida, United States<br>•Northwestern University, Chicago, Illinois, United States<br>•Siouxland Regional Cancer Center, Sioux City, Iowa, United States<br>•University of Michigan Comprehensive Cancer Center, Ann Arbor, Michigan, United States<br>•Mayo Clinic, Rochester, Minnesota, United States<br>•Nebraska Methodist Hospital, Omaha, Nebraska, United States<br>•Memorial Sloan Kettering Basking Ridge, Basking Ridge, New Jersey, United States<br>•Memorial Sloan Kettering Monmouth, Middletown, New Jersey, United States<br>•Memorial Sloan Kettering Bergen, Montvale, New Jersey, United States<br>•Memorial Sloan Kettering Commack, Commack, New York, United States<br>•and 11 more
73	Study of Sorafenib/Cetuximab in Head and Neck Cancer	Completed	Has Results	•Squamous Cell Cancer	•Drug: Sorafenib<br>•Drug: Cetuximab	•Duke University Health System, Durham, North Carolina, United States
74	Study of Combination of Metronomic Oral Vinorelbine and Sorafenib in Patients With Advanced Non-small Cell Lung Cancer	Completed	No Results Available	•Non-Small Cell Lung Cancer	•Drug: oral vinorelbine<br>•Drug: sorafenib	•National Cancer Centre, Singapore, Singapore, Singapore

	Title	Status	Study Results	Conditions	Interventions	Locations
75	Phase 2b Study of Taxol Plus Sorafenib or Placebo in Patients With Advanced Breast Cancer	Active, not recruiting	No Results Available	•Breast Cancer	•Drug: paclitaxel<br>•Drug: sorafenib tosylate<br>•Other: placebo	•Northwest Alabama Cancer Center, PC - Muscle Shoals, Muscle Shoals, Alabama, United States<br>•Highlands Oncology Group - Fayetteville, Fayetteville, Arkansas, United States<br>•Pacific Cancer Medical Center, Incorporated, Anaheim, California, United States<br>•Pacific Coast Hematology/Oncology Medical Group, Incorporated, Fountain Valley, California, United States<br>•Desert Hematology-Oncology Medical Group, Incorporated, Rancho Mirage, California, United States<br>•Sutter Cancer Center, Sacramento, California, United States<br>•Saint Helena, California, United States<br>•Cancer Prevention and Treatment Center at Dominican and Watsonville Community Hospital, Soquel, California, United States<br>•Helen and Harry Gray Cancer Center at Hartford Hospital, Hartford, Connecticut, United States<br>•Eastern Connecticut Hematology and Oncology Associates, Norwich, Connecticut, United States<br>•and 33 more
76	Gemcitabine With or Without Sorafenib in Treating Patients With Locally Advanced or Metastatic Pancreatic Cancer	Unknown status	No Results Available	•Pancreatic Cancer	•Drug: gemcitabine hydrochloride<br>•Drug: sorafenib tosylate	•Marseille Institute of Cancer - Institut J. Paoli and I. Calmettes, Marseille, France
77	Sorafenib and High-Dose Carboplatin, Paclitaxel, and External-Beam Radiation Therapy in Treating Patients With Stage III Non-Small Cell Lung Cancer	Withdrawn	No Results Available	•Lung Cancer	•Drug: carboplatin<br>•Drug: paclitaxel<br>•Drug: sorafenib tosylate<br>•Radiation: radiation therapy	•Arlington Cancer Center - Arlington, Arlington, Texas, United States<br>•Simmons Comprehensive Cancer Center at University of Texas Southwestern Medical Center - Dallas, Dallas, Texas, United States
78	Sorafenib Tosylate With or Without Everolimus in Treating Patients With Localized, Unresectable, or Metastatic Liver Cancer	Completed	No Results Available	•Liver Cancer	•Drug: everolimus<br>•Drug: sorafenib tosylate	•Medizinische Universität Wien, Wien, Austria<br>•Szent Laszlo Korhaz, Budapest, Hungary<br>•Saint Claraspital AG, Basel, Switzerland<br>•Clinical Cancer Research Center at University Hospital Basel, Basel, Switzerland<br>•Istituto Oncologico della Svizzera Italiana - Ospedale Regionale Bellinzona e Valli, Bellinzona, Switzerland<br>•Inselspital Bern, Bern, Switzerland<br>•Kantonsspital Bruderholz, Bruderholz, Switzerland<br>•Hopital Cantonal Universitaire de Geneve, Geneva, Switzerland<br>•Centre Hospitalier Universitaire Vaudois, Lausanne, Switzerland<br>•Kantonsspital Liestal, Liestal, Switzerland<br>•and 5 more
79	Sorafenib in Treating Patients With Locally Advanced or Metastatic Liver Cancer and Cirrhosis	Terminated	No Results Available	•Liver Cancer	•Drug: sorafenib tosylate	•Lineberger Comprehensive Cancer Center at University of North Carolina - Chapel Hill, Chapel Hill, North Carolina, United States<br>•Duke Comprehensive Cancer Center, Durham, North Carolina, United States

	Title	Status	Study Results	Conditions	Interventions	Locations
80	Sorafenib/Erlotinib Versus Erlotinib Alone in Previously Treated Advanced Non-Small-Cell Lung Cancer (NSCLC)	Completed	Has Results	•Non-Small Cell Lung Cancer	•Drug: Erlotinib + Sorafenib<br>•Drug: Erlotinib + Placebo	•Florida Cancer Specialists, Fort Myers, Florida, United States<br>•Northeast Georgia Medical Center, Gainesville, Georgia, United States<br>•Wellstar Cancer Research, Marietta, Georgia, United States<br>•Kansas City Cancer Centers, Overland Park, Kansas, United States<br>•Center for Cancer and Blood Disorders, Bethesda, Maryland, United States<br>•Grand Rapids Clinical Oncology Program, Grand Rapids, Michigan, United States<br>•Methodist Cancer Center, Omaha, Nebraska, United States<br>•Cancer Care of Western North Carolina, Asheville, North Carolina, United States<br>•Oncology Hematology Care, Cincinnati, Ohio, United States<br>•Mid Ohio Oncology/Hematology, Inc./ The Mark H. Zangmeister Center, Columbus, Ohio, United States<br>•and 6 more
81	BAY 43-9006 in Previously Untreated Patients With Non-Small Cell Lung Cancer (NSCLC)	Completed	No Results Available	•Lung Cancer	•Drug: BAY 43-9006<br>•Drug: Paclitaxel<br>•Drug: Carboplatin<br>•Drug: Bevacizumab	•University of Texas MD Anderson Cancer Center, Houston, Texas, United States
82	Evaluate Safety and Tolerability in Dose Escalation of Sorafenib in Advanced Renal Cell Cancer	Completed	Has Results	•Renal Cell Cancer	•Drug: sorafenib	•University of Kansas Medical Center, Kansas City, Kansas, United States
83	Pemetrexed Disodium With or Without Sorafenib as Second-Line Therapy in Treating Patients With Stage IIIB or Stage IV Non-Small Cell Lung Cancer	Completed	Has Results	•Lung Cancer	•Drug: pemetrexed disodium<br>•Drug: sorafenib tosylate	•Mayo Clinic Scottsdale, Scottsdale, Arizona, United States<br>•Aurora Presbyterian Hospital, Aurora, Colorado, United States<br>•Boulder Community Hospital, Boulder, Colorado, United States<br>•Penrose Cancer Center at Penrose Hospital, Colorado Springs, Colorado, United States<br>•St. Anthony Central Hospital, Denver, Colorado, United States<br>•Porter Adventist Hospital, Denver, Colorado, United States<br>•Presbyterian - St. Luke's Medical Center, Denver, Colorado, United States<br>•St. Joseph Hospital, Denver, Colorado, United States<br>•Rose Medical Center, Denver, Colorado, United States<br>•CCOP - Colorado Cancer Research Program, Denver, Colorado, United States<br>•and 197 more

	Title	Status	Study Results	Conditions	Interventions	Locations
84	Sorafenib + Topotecan for Platinum-Resistant Recurrent Ovarian Cancer	Terminated	Has Results	•Ovarian Cancer	•Drug: Sorafenib<br>•Drug: Topotecan	•Medical & Surgical Specialists, LLC, Galesburg, Illinois, United States<br>•Oncology Hematology Associates of SW Indiana, Evansville, Indiana, United States<br>•Fort Wayne Oncology & Hematology, Inc, Fort Wayne, Indiana, United States<br>•Indiana University Simon Cancer Center, Indianapolis, Indiana, United States<br>•St. Vincent Hospital Cynecologic Oncology, Indianapolis, Indiana, United States<br>•Arnett Cancer Care, Lafayette, Indiana, United States<br>•Medical Consultants, P.C., Muncie, Indiana, United States<br>•Schwartz Gynecologic Oncology, PLLC, Brightwaters, New York, United States
85	2nd Line Treatment With Pemetrexed and Sorafenib for Recurrent or Metastatic Triple Negative Breast Cancer	Terminated	Has Results	•Breast Cancer<br>•Metastatic Breast Cancer<br>•Recurrent Breast Cancer	•Drug: Experimental Arm A: Pemetrexed<br>•Drug: Experimental Arm A: Sorafenib<br>•Drug: Experimental Arm B: Pemetrexed<br>•Drug: Experimental Arm B: Sorafenib	•Virginia Commonwealth University/Massey Cancer Center, Richmond, Virginia, United States
86	Study of Gemcitabine and Erlotinib Plus Sorafenib (GES) in Metastatic Pancreatic Cancer	Completed	Has Results	•Pancreatic Cancer	•Drug: Gemcitabine<br>•Drug: Erlotinib<br>•Drug: Sorafenib	•Desert Regional Medical Center, Palm Springs, California, United States<br>•Bellevue Hospital, New York, New York, United States<br>•New York University Cancer Center, New York, New York, United States
87	A Phase II Study Of Abraxane and Nexavar in the First-Line Treatment of Locally Advanced or Metastatic Breast Cancer	Terminated	No Results Available	•Breast Cancer	•Drug: Paclitaxel Albumin Nanoparticle for Injectable Suspension (Abraxane)<br>•Drug: Sorafenib (Nexavar)	•Veeda Oncology, Columbus, Ohio, United States
88	Sorafenib in Treating Patients With Extensive Stage Small Cell Lung Cancer	Completed	Has Results	•Extensive Stage Small Cell Lung Cancer<br>•Recurrent Small Cell Lung Cancer	•Drug: sorafenib tosylate	•SWOG, Portland, Oregon, United States
89	Phase I Trial of Bevacizumab and Temsirolimus in Combination With 1) Carboplatin, 2) Paclitaxel, 3) Sorafenib for the Treatment of Advanced Cancer	Active, not recruiting	No Results Available	•Advanced Cancer	•Drug: Temsirolimus<br>•Drug: Bevacizumab<br>•Drug: Paclitaxel<br>•Drug: Sorafenib<br>•Drug: Carboplatin	•University of Texas MD Anderson Cancer Center, Houston, Texas, United States
90	Activity of Sorafenib in Salivary Gland Cancer	Completed	No Results Available	•Salivary Gland Cancer	•Drug: Sorafenib	•IRCCS Istituto Nazionale Tumori, Milano, Italy
91	Phase I Study of Weekly Topotecan in Combination With Sorafenib in Treatment of Relapsed Small Cell Lung Cancer	Completed	No Results Available	•Small Cell Carcinoma<br>•Lung Cancer	•Drug: Topotecan<br>•Drug: Sorafenib	•Park Nicollet Institute, St. Louis Park, Minnesota, United States
92	The Effects of Sorafenib on Molecular Barrett's Esophagus Cancer	Terminated	Has Results	•Esophageal Cancer	•Drug: sorafenib	•University of Chicago, Chicago, Illinois, United States

	Title	Status	Study Results	Conditions	Interventions	Locations
93	Whole Brain Radiotherapy (WBRT) With Sorafenib for Breast Cancer Brain Metastases (BCBM)	Completed	No Results Available	•Breast Cancer<br>•Brain Metastases	•Radiation: Whole Brain Radiotherapy (WBRT)<br>•Drug: Sorafenib	•Memoral Sloan Kettering Cancer Center, Basking Ridge, New Jersey, United States<br>•Memorial Sloan Kettering Monmouth, Middletown, New Jersey, United States<br>•Memorial Sloan Kettering Cancer Center @ Suffolk, Commack, New York, United States<br>•Memorial Sloan Kettering Westchester, Harrison, New York, United States<br>•Memorial Sloan Kettering Cancer Center, New York, New York, United States<br>•Memorial Sloan Kettering Nassau, Uniondale, New York, United States
94	Sorafenib Plus Toripalimab for Unresectable HCC With Portal Vein Tumor Thrombus	Unknown status	No Results Available	•Unresectable Hepatocellular Cancer<br>•Portal Vein Tumor Thrombus	•Drug: sorafenib; toripalimab	
95	Sorafenib and Bevacizumab in Combination With Paclitaxel in Patients With Solid Tumors	Completed	No Results Available	•SOLID TUMORS	•Drug: Sorafenib	•Indiana University Cancer Center, Indianapolis, Indiana, United States
96	Sorafenib in Treating Patients With Metastatic or Unresectable Kidney Cancer	Terminated	Has Results	•Kidney Cancer	•Drug: Sorafenib	•UNMC Eppley Cancer Center at the University of Nebraska Medical Center, Omaha, Nebraska, United States
97	Sorafenib Tosylate With or Without Pravastatin in Treating Patients With Liver Cancer and Cirrhosis	Completed	No Results Available	•Liver Cancer	•Drug: pravastatin sodium<br>•Drug: sorafenib tosylate	•Hopital Du Bocage, Dijon, France
98	Sorafenib in Treating Patients With Metastatic Kidney Cancer That Has Not Responded to Sunitinib or Bevacizumab	Completed	Has Results	•Kidney Cancer	•Drug: sorafenib tosylate	•Cleveland Clinic Taussig Cancer Institute, Case Comprehensive Cancer Center, Cleveland, Ohio, United States<br>•Baylor Sammons Cancer Center, Dallas, Texas, United States
99	Sorafenib in Treating Patients Undergoing Surgery for Stage II, Stage III, or Stage IV Kidney Cancer	Completed	No Results Available	•Kidney Cancer	•Drug: sorafenib tosylate	•Lineberger Comprehensive Cancer Center at University of North Carolina - Chapel Hill, Chapel Hill, North Carolina, United States
100	Sorafenib and FOLFIRI Regimen in 2nd Colorectal Cancer (CRC) After Failure of Oxaliplatin Treatment	Unknown status	No Results Available	•Colorectal Neoplasms	•Drug: sorafenib<br>•Drug: FOLFIRI	•Cancer Hospital,Fudan University, Shanghai, Shanghai, China

790 additional studies not shown
