# Supplementary material for: Thymoquinone, piperine, and sorafenib combinations attenuate liver and breast cancers progression: epigenetic and molecular docking approaches
Source: BMC Complement Med Ther. 2023 Mar 4;23:69. doi: 10.1186/s12906-023-03872-6 (PMC9985300; doi:10.1186/s12906-023-03872-6)
Supplement: Supplementary file 4 — Additional file 4. Thymoquinone anticancer clinical trials. [file 12906_2023_3872_MOESM4_ESM.pdf]

Supplementary File 4: Thymoquinone anticancer clinical trials

ClinicalTrials.gov Search Results 07/27/2022

	Title	Status	Study Results	Conditions	Interventions	Locations
1	Clinical and Immunohisochemical Evaluation of Chemopreventive Effect of Thymoquinone on Oral Potentially Malignant Lesions.	Completed	No Results Available	•Premalignant Lesion	•Drug: Nigella sativa buccal tablets 10mg<br>•Drug: Nigella sativa buccal tablets 5mg<br>•Drug: Placebo buccal tablets	•Faculty of Dentistry, Cairo, Egypt
2	Amelioration of Polycystic Ovary Syndrome Related Disorders by Supplementation of Thymoquinone and Metformin	Completed	No Results Available	•Polycystic Ovary Syndrome (PCOS)	•Drug: Metformin Versus a combination of Metformin and Thymoquinone (TQ)	•Saudi German Hospital, Al Mad#nah, Madinah, Saudi Arabia

U.S. National Library of Medicine | U.S. National Institutes of Health | U.S. Department of Health & Human Services
