# Supplementary material for: Thymoquinone, piperine, and sorafenib combinations attenuate liver and breast cancers progression: epigenetic and molecular docking approaches
Source: BMC Complement Med Ther. 2023 Mar 4;23:69. doi: 10.1186/s12906-023-03872-6 (PMC9985300; doi:10.1186/s12906-023-03872-6)
Supplement: Supplementary file 6 — Additional file 6. Piperine anticancer clinical trials. [file 12906_2023_3872_MOESM6_ESM.pdf]

Supplementary File 6: Piperine anticancer clinical trials

ClinicalTrials.gov Search Results 07/27/2022

	Title	Status	Study Results	Conditions	Interventions	Locations
1	Curcumin and Piperine in Reducing Inflammation for Ureteral Stent-Induced Symptoms in Patients With Cancer	Active, not recruiting	No Results Available	• Bladder Spasm<br>• Malignant Neoplasm<br>• Pain<br>• Urinary Urgency	• Drug: Curcumin<br>• Other: Laboratory Biomarker Analysis<br>• Dietary Supplement: Piperine Extract (Standardized)<br>• Other: Quality-of-Life Assessment	• Mayo Clinic, Rochester, Minnesota, United States
2	Curcumin and Piperine in Patients on Surveillance for Monoclonal Gammopathy, Smoldering Myeloma or Prostate Cancer	Recruiting	No Results Available	• Prostate Cancer<br>• Multiple Myeloma<br>• Smoldering Multiple Myeloma (SMM)<br>• Monoclonal Gammopathy of Undetermined Significance	• Drug: Curcumin plus Piperine	• University of Rochester, Rochester, New York, United States

U.S. National Library of Medicine | U.S. National Institutes of Health | U.S. Department of Health & Human Services
